# Catalysis by framework zinc in silica-based molecular sieves†
†Electronic supplementary information (ESI) available. See DOI: 10.1039/c5sc03889h


**DOI:** 10.1039/c5sc03889h

**Published:** 2016-01-04

**Authors:** Marat Orazov, Mark E. Davis

**Affiliations:** a Chemical Engineering , California Institute of Technology , Pasadena , 91125 , USA . Email: mdavis@cheme.caltech.edu

## Abstract

Microporous and mesoporous zincosilicates (*e.g.*, CIT-6, VPI-8, Zn-MFI, and Zn-MCM-41) synthesized in the presence of alkali cations contain two broad types of Zn sites: one that is a dication analog of the monocation ion-exchangeable Al-site in aluminosilicates, while the other resembles isolated Zn sites on amorphous silica. The ratio of these sites varies, depending on the synthesis conditions of the zincosilicate. Post-synthetic strategies based on ion-exchange can alter the site distribution towards either population. Furthermore, post-synthetic introduction of isolated Zn sites of the latter type is possible for materials possessing silanol nests. Both types of sites behave as Lewis acid centers in probe-molecule IR spectroscopy, but have very different catalytic properties. Due to the unusually high adsorption energies of Lewis bases on such materials, Lewis acid catalysis is difficult at low temperatures and in solvents bearing Lewis basic functionality. However, at high temperatures, in hydrocarbon solvents, CIT-6 (Zn-beta) is able to selectively catalyze the Lewis-acid-catalyzed Diels–Alder cycloaddition–dehydration reactions of ethylene with methyl 5-(methoxymethyl)furan-2-carboxylate, a furan that can be derived quantitatively by partial oxidation of biomass-based 5-hydroxymethylfurfural. Additionally, zinc in silica-based molecular sieves is shown here to enable chemistries previously not accessible with framework Sn-, Ti- and Zr-based Lewis acid sites, *e.g.*, the direct production of dimethyl terephthalate by Diels–Alder cycloaddition–dehydration reactions of ethylene and the dimethyl ester of furan-2,5-dicarboxilic acid.

## Abstract 2

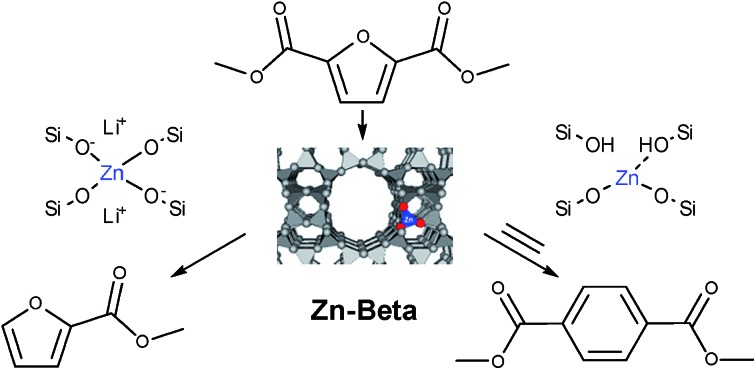
Adjustable Zn Lewis acid site distributions in crystalline, microporous zincosilicates were studied spectroscopically and explored for catalytic applications.

## 1. Introduction

Heterogeneous catalysts consisting of isolated Lewis acid centers on silica-based supports have been investigated for a wide range of reactions for the conversion of biomass into valorized chemicals. Generation of framework, Lewis acid sites in crystalline, pure-silica molecular sieves by the isomorphic substitution of Si by Sn, Ti, Zr, or Hf is particularly interesting because such sites are located in pores that have diameters comparable to those of substrates, thus giving rise to the possibility of shape-selective catalysis and support-induced stabilization of transition states or intermediates.^1^–^5^ These materials can also exhibit higher acid site stability, with lower tendency for thermal ion migration and sintering into bulk oxides, than analogous sites on amorphous supports. The crystalline materials have been shown to be catalytically active in alkane oxidation, alkene epoxidation, aromatics hydroxylation, Baeyer–Villiger (BV) oxidation, Meerwein–Ponndorf–Verley–Oppenauer (MPVO), sugar isomerization, retro-aldol, and Diels–Alder cycloaddition–dehydration reactions and can, in some instances, be coupled with other catalytic chemistries in “one-pot” strategies.^1^,^6^–^15^ The catalytic performance of these materials appears to depend strongly on the heteroatom type, the framework in which they are located, and the particular reaction conditions utilized. No single heterogeneous Lewis acid catalyst has been shown to offer optimal performance for the broad range of reactions that are believed to involve Lewis acid activation. Thus, discovery and characterization of catalytically active sites for given reactions is necessary to guide process optimization, and must be performed on an individual basis. Expanding the number of members in this library of zeotypic catalysts is an ongoing effort in the field, with attempts being made to both increase the number of usable zeolitic frameworks, and enable the incorporation of catalytically pertinent metal centers in a controlled manner.

Zinc is a common metal center in a number of homogeneous, Lewis acid catalysts (both in synthetic complexes and naturally-occurring enzymes).^16^–^18^ While Zn^2+^ ions exchanged onto aluminosilicate zeolites have been considered for a number of catalytic applications, to the best of our knowledge, evidence of heterogeneous catalysis performed by framework Zn sites in otherwise pure-silica molecular sieves is sparse.^19^,^20^ For instance, Zn^2+^ exchanged onto Al-beta preferentially titrates paired Al sites, and the resulting material can be used as a catalyst for hydroamination reactions.^19^ Such Zn^2+^ sites have been extensively characterized and were demonstrated to have very strong interactions with Lewis basic probe molecules such as acetonitrile and pyridine, with characteristic desorption temperatures considerably higher than those observed in our previous studies of Lewis acidic zeotypes containing Ti, Zr, or Sn.^19^,^21^ However, in all such samples, residual Brønsted acidity, originating from isolated Al sites that are not readily exchanged by Zn^2+^, is observed.^19^ These results, as well as literature on amorphous silica materials bearing isolated Zn sites,^22^–^24^ have prompted us to investigate the properties of CIT-6, an easily-synthesized zincosilicate analog of zeolite beta, whose Lewis and Brønsted acidity have not been sufficiently characterized, despite its first reported synthesis dating back to 1999.^25^,^26^ Instead of direct catalysis by Zn sites, to date, CIT-6 has been mainly used as a support for other metal centers, *e.g.*, Ni^2+^ or Pt^2+^ ions exchanged onto the zincosilicate, or aluminum inserted into the silanol nests of its de-zincated form.^25^,^27^,^28^ Here, we report our characterization of CIT-6 and other similar zincosilicates by probe-molecule FTIR spectroscopy and evaluate their catalytic properties in the context of Lewis acid mediated reactions, namely: isomerization of glucose to fructose, MPVO reactions of cyclohexanone and 2-butanol, and Diels–Alder cycloaddition–dehydration reactions of partially oxidized variants of 5-hydroxymethylfurfural (5-HMF). We show that such Zn zeotypes have exceedingly strong interactions with Lewis basic substrates. While such high interaction strengths can limit the conditions where catalysis is feasible, under appropriate conditions, these materials may enable chemistries that previously were effectively inaccessible, *e.g.*, Diels–Alder cycloaddition–dehydration reactions of the dimethyl ester of furan-2,5-dicarboxylic acid.

## 2. Experimental

### 2.1. Microporous materials synthesis

The syntheses of microporous and mesoporous materials used in this study are standard and are reported elsewhere, but are briefly outlined below. In each case, as-synthesized solids were recovered by centrifugation, washed thoroughly with distilled water and acetone (Fisher Scientific), dried at 100 °C, and calcined in 100 mL min^–1^ flowing air (Air Liquide, breathing grade) at 580 °C (ramped up at 1 °C min^–1^) for 6 h.

#### 2.1.1. CIT-6 synthesis

CIT-6 was synthesized according to the method reported by Takewaki *et al.*^25^ Colloidal silica (Ludox AS-40), zinc acetate dihydrate (Aldrich), tetraethylammonium hydroxide (Aldrich), and lithium hydroxide monohydrate (Aldrich) were mixed to form a clear synthesis gel of composition 1SiO_2_/0.03Zn(OAc)_2_/0.65TEAOH/0.05LiOH/30H_2_O. The gel was charged into a Teflon-lined, stainless steel autoclave and heated statically at 140 °C for 7.5 days under autogenous pressure. A single large batch (8 g SiO_2_ in gel) of the material was synthesized and used throughout the study.

#### 2.1.2. VPI-8 synthesis

VPI-8 was synthesized from the same gel as CIT-6, above, but at a higher temperature (150 °C) and longer crystallization times (14 days).

#### 2.1.3. Zn-MFI synthesis

Zn-MFI was synthesized according to the method reported by BP.^29^ Zinc sulfate heptahydrate (Aldrich) was dissolved in distilled water and the pH was increased to 6 by addition of ammonia solution (Mallinckrodt). The white precipitate that formed (presumably Zn(OH)_2_) was filtered, washed thoroughly with distilled water, and dried. Sodium hydroxide (Mallinckrodt) and TPAOH (Acros Organics) dissolved in distilled water were added to the precipitate and stirred until the precipitate dissolved. Ludox AS-40 was added to the solution with stirring. The resulting homogeneous gel of composition 1SiO_2_/0.067ZnO/0.105TPAOH/0.107Na_2_O/14.6H_2_O was charged into a Teflon-lined, stainless steel autoclave and heated in a rotating oven at 175 °C for 4 days under autogenous pressure.

#### 2.1.4. Zn-MCM-41 synthesis

Zn-MCM-41 was synthesized according to the method reported by Takewaki *et al.*^30^ Tetra-ethylorthosilicate (Aldrich), zinc acetate dihydrate, cetyltrimethylammonium bromide (Aldrich), and sodium hydroxide were mixed at ambient temperature for 2 h to form a gel of composition 1SiO_2_/0.02Zn(OAc)_2_/0.61C_16_TMABr/0.5NaOH/4EtOH/30H_2_O. The gel was charged into a Teflon-lined, stainless steel autoclave and heated statically at 105 °C for 3 days under autogenous pressure.

#### 2.1.5. SSZ-33 synthesis

The borosilicate SSZ-33 was synthesized according to the method reported by Dartt and Davis.^9^ Fumed silica (Cab-O-Sil), boric acid (J. T. Baker), *N*,*N*,*N*-trimethyltricyclo[5.2.1.0^2,6^] decaneammonium–hydroxide (R–OH) (provided by Dr Stacey I. Zones of Chevron Energy Technology Company), and sodium hydroxide were mixed for 1 h to form a synthesis gel of composition 1SiO_2_/0.0125B_2_O_3_/0.2ROH/0.1NaOH/40H_2_O. The gel was charged into a Teflon-lined, stainless steel autoclave and heated in a rotating oven at 160 °C for 10 days under autogenous pressure.

#### 2.1.6. Zr-beta synthesis

Zr-beta was synthesized in fluoride media according to the methods adopted by Pacheco and Davis.^13^ Tetraethylorthosilicate was partially hydrolysed in a solution of tetraethylammonium hydroxide for 30 min, followed by the addition of zirconium(iv) propoxide (Aldrich) in ethanol. The alkoxides were allowed to hydrolyse overnight and excess water and alcohols were evaporated. Finally hydrofluoric acid (HF) (Aldrich) was added to form a synthesis gel of composition 1SiO_2_/0.1ZrO_2_/0.54TEAOH/0.54HF/6.75H_2_O. Dealuminated Al-beta seeds (prepared according to the method reported by Chang *et al.*)^31^ in water were dispersed in the Zr-beta gel prior to crystallization (at 4 wt% loading of SiO_2_). The gels was charged into a Teflon-lined, stainless steel autoclave and heated in a rotating oven at 140 °C for 7 days under autogenous pressure.

#### 2.1.7. Generation of silanol nests by heteroatom removal

Zn was inserted into some materials that contained silanol nests generated by removal of Zn or B by treatment of calcined zeolite powders with 1 M aqueous H_2_SO_4_ (Macron) (at 0.1 g solid/10 mL solution) at ambient temperatures for 12 h. The final solids were recovered by centrifugation, thoroughly washed and calcined prior to further use.

#### 2.1.8. Post-synthetic Zn insertion

The zinc insertion procedure was adapted from Kozawa.^24^ Material possessing silanols were contacted with an aqueous solution of 0.1 M ZnCl_2_ (EM Science), and 2.0 M NH_4_Cl (Mallinckrodt) (adjusted to desired pH by NH_4_OH or HCl), using 1 g solid/25 mL solution. The dispersed solids were stirred in such solutions for 12 h at ambient temperature.

Na/Zn/Al-beta and Zn/Al-beta were generated by adapting the Zn-exchange procedure of Penzien *et al.*^19^ 0.06 M Zn(OAc)_2_ aqueous solution was contacted with calcined Na–Al-beta or H–Al-beta (Tosoh), at 80 °C, for 24 h, using 1 g solid/25 mL solution.

After the exchange procedures, the solids were recovered by centrifugation, washed twice with distilled water (1 g solid/25 mL water), dried, and calcined, as previously described.

### 2.2. Characterization of solids

Scanning electron microscopy (SEM) with energy dispersive X-ray spectroscopy (EDS) measurements were recorded on a LEO 1550 VP FE SEM at an electron high tension (EHT) of 15 kV. The crystalline structures of zeolite samples were determined from powder X-ray diffraction (XRD) patterns collected using a Rigaku Miniflex II diffractometer and Cu Kα radiation. Thermogravimetric analysis (TGA) under an air atmosphere was performed on a PerkinElmer STA 6000 with a ramp of 10 °C min^–1^ up to 900 °C. Probe molecule IR spectroscopy experiments were performed on a Nicolet Nexus 470 Fourier transform infrared (FTIR) spectrometer with a liquid N_2_ cooled Hg–Cd–Te (MCT) detector. Spectra in 4000–650 cm^–1^ range were acquired with 2 cm^–1^ resolution. Self-supporting wafers (10–20 mg cm^–2^) were pressed and sealed in a heatable quartz vacuum cell with removable KBr windows. The cell was purged with air (60 mL min^–1^, Air Liquide, breathing grade) while heating to 500 °C (1 °C min^–1^), where it was held for 1 h, followed by evacuation at 500 °C for >2 h (<0.01 Pa dynamic vacuum; oil diffusion pump), and cooling to 35 °C under a dynamic vacuum. CD_3_CN (Sigma-Aldrich, 99.8% D atoms) or pyridine (EMD Millipore) was degassed by three freeze (liquid N_2_), pump, thaw cycles, then dosed to the sample at 35 °C until the Lewis acid sites were saturated, at which point physisorbed and gas-phase species were observed. The cell was evacuated down to 13.3 Pa, and the first spectrum was recorded. Then, the cell was evacuated under a dynamic vacuum at 35 °C for 24 h, after which the second spectrum was acquired. Subsequent heating to temperatures specified in plots of spectra was performed at 5 °C min^–1^, with a subsequent hold time of 0.5 h or greater (as specified in figure legends) prior to acquisition of the next spectrum. The resulting spectra were baseline-corrected by subtracting the spectrum of the pellet at same temperature prior to adsorption of the probe molecule. The spectra are not normalized by the number of Lewis acid sites or framework vibrations. Spectral artifacts known as “interference fringes” were apparent in some spectra and were removed using a computational method based on digital filtering techniques and Fourier analysis.^32^

### 2.3. Catalytic testing

Liquid ^1^H and ^13^C NMR spectra were acquired on a Varian 500 MHz spectrometer equipped with an auto-x pfg broadband probe and a Bruker 400 MHz with Prodigy broadband cryoprobe. Carbohydrate analysis was performed *via* high performance liquid chromatography on an Agilent 1200 system equipped with refractive index and evaporative light scattering detectors. An Agilent Hi-Plex Ca column at 80 °C was used with ultrapure water as the mobile phase (flow rate of 0.6 mL min^–1^). Quantitative GC-FID analysis of MPVO and DA cycloaddition dehydration reactions was performed on an Agilent 7890B GC system equipped with a flame ionization detector and an Agilent HP-5 column. Qualitative GC-MS analysis of products was performed on an Agilent 5890 GC system with an Agilent 5970 mass spectrometer and an Agilent DB-5 column.

#### 2.3.1. Glucose isomerization reactions

Reactions of glucose (Aldrich) (1 wt% in water or methanol (EMD Millipore) solvent) catalysed by CIT-6 were performed in 10 mL thick-walled crimp-sealed glass reactors (VWR) that were heated in a temperature-controlled oil bath. Reactions were performed at 100 °C, with a 1 : 50 Zn : glucose initial molar ratio. Aliquots (∼100 μL) were extracted at indicated times, mixed with a mannitol solution (external standard), filtered with a 0.2 μm PTFE syringe filter, and analysed by HPLC. To determine the mechanism of fructose formation, glucose ^13^C-enriched at the C1 position (^13^C-C1-glucose) (Cambridge Isotope) was reacted with CIT-6 in D_2_O at 100 °C for 1 h. The product solution was filtered and analysed by liquid NMR directly.

#### 2.3.2. MPVO reactions

Reactions of cyclohexanone (Aldrich) and 2-butanol (Fisher Scientific) catalysed by CIT-6 were performed in 10 mL thick-walled crimp-sealed glass reactors (VWR) that were heated in a temperature-controlled oil bath. Reactions were performed at 100 °C, in cyclohexane solvent. The cyclohexanone concentration was fixed at 0.1 M for all reactions, and the initial ratio of Zn : cyclohexanone was 1 : 100. Naphthalene was used as an internal standard, and reactions were analysed by GC-FID. The turnover frequency at a given 2-butanol concentration was calculated from the initial rate of formation of cyclohexanol.

#### 2.3.3. Diels–Alder reactions

The procedure for Diels–Alder reactions was adapted from Pacheco and Davis,^13^ but was modified for quantification by GC-FID. Reactions of methyl 5-(methoxymethyl)furan-2-carboxylate (MMFC) (enamine) or dimethyl 2,5-furandicarboxylate (DMFDC) (Matrix Scientific) with ethylene (Matheson) were carried out in a 50 mL high-pressure stainless steel batch reactor (Parr Series 4590) equipped with a magnetic stirrer (operated at 200 rpm) and heater. For MMFC reactions, 10 mL of a 0.1 M diene solution in heptane (Aldrich) and 100 mg catalyst were loaded into the reactor. DMFDC is poorly soluble in heptane at low temperatures, so this diene, along with 100 mg catalyst were loaded directly into the reactor, and 10 mL of heptane was added to give a nominal concentration of 0.33 M of diene. Decane was also added as an internal standard for GC-FID quantification. At the start of a reaction, the head space of the reactor was purged with helium gas with a fill/vent cycle (10 times). Next, the reactor was pressurized to 35 bar with ethylene gas at ambient temperature. The reactor was heated to reaction temperature while the pressure increased autogenously (∼60–80 bar). Reaction time was started when the contents of the vessel reached desired temperature, and after a specified time, the reactor was quenched with water and allowed to cool to ambient temperature. At this point, the reactor gases were carefully vented. Solution aliquots that were collected for GC analysis were filtered with a 0.2 μm PTFE syringe filter. The MMFC reaction solutions were analysed directly, while for the DMFDC system, 20 mL of acetone were added to solubilize components not readily soluble in heptane. In both cases, aliquots taken for NMR studies were filtered, rotavaped, and redissolved in acetone-d_6_ (Cambridge Isotope). Samples of post-reaction catalysts intended for TGA were isolated from reaction solution by centrifugation, washed twice with either heptane (for MMFC reactions) or acetone (for DMFDC reactions), and dried at 100 °C overnight. TGA of corresponding washed, unreacted catalysts was used to account for any strongly-retained solvent.

Catalyst recycle experiments were performed for the Diels–Alder cycloaddition–dehydration reaction of DMFDC and ethylene at 210 °C, with CIT-6-reZn-pH = 6.9. First two reruns were performed with samples that were triply washed with acetone and dried at 100 °C overnight. The third rerun was performed on recalcined catalyst recovered after the second rerun. In all instances reagent and solvent ratios were adjusted to keep constant ratio to inorganic content, as determined by TGA. SEM-EDS analysis of recovered and acetone-washed catalysts was performed. The catalyst used in the third rerun was also analysed by XRD and CD_3_CN adsorption tracked by IR.

## 3. Results and discussion

### 3.1. Probe molecule FTIR spectroscopy

The presence and character of Lewis and Brønsted acid sites in solid catalysts may be probed by following the adsorption and desorption behavior of Lewis basic molecules through FTIR spectroscopy. Pyridine and deuterated acetonitrile (CD_3_CN) have been routinely used as Lewis bases for this purpose.^33^,^34^ While the significantly different vibrational modes of pyridine coordinated to a Lewis acid center and pyridinium ion generated from protonation of pyridine by a Brønsted acid allow for easy determination of the presence of the two kinds of sites, more subtle differences that differentiate one kind of Lewis acid center from another are harder to discern. Thus, CD_3_CN, whose CN stretching frequency tends to increase with the strength of the interaction with a Lewis acid center, can be used as a complimentary probe molecule to qualitatively compare the site of interest with other previously studied sites.^33^ Additionally, desorption temperatures of such molecules reflect the strength of interactions a given functional group may be expected to have with the site of interest.

**Fig. 1:**
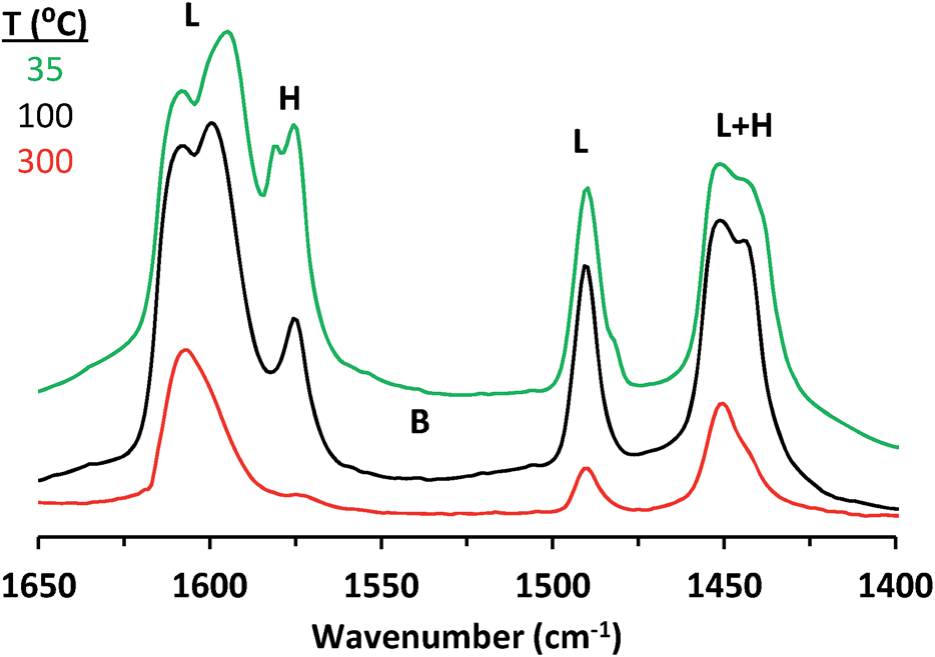
Baseline corrected IR spectra of pyridine adsorbed on CIT-6 at 35 °C. Different colors (indicated in legend) correspond to different subsequent desorption temperatures carried out for 1 h. Peaks corresponding to pyridine coordinated to Lewis acid (L), Brønsted acid (B), and hydrogen-bonding (H) sites are marked.

Pyridine adsorption onto CIT-6 (Fig. 1) results in IR bands characteristic of pyridine interacting with Lewis acid sites (1451, 1491, and 1610 cm^–1^) and hydrogen bonded pyridine (*ca.* 1575 and 1446 cm^–1^).^23^ No band characteristic of Brønsted acid sites (*ca.* 1550 cm^–1^) is observed. Pyridine remains adsorbed on the Lewis acid sites up to 300–350 °C. In contrast, pyridine dosed onto bulk ZnO results in bands characteristic of Lewis acid sites (1451 and 1610 cm^–1^), hydrogen-bonded pyridine (1574 cm^–1^), and a broad band in the range previously assigned to both dissociatively-adsorbed pyridine on base sites (C_5_H_4_N^–^ species) and to protonated pyridine on strong Brønsted acid sites (C_5_H_5_NH^+^ species)^33^,^34^ (Fig. S1†). Furthermore, all such adsorbed pyridine desorbs upon evacuation at 300 °C (Fig. S1†).

**Fig. 2:**
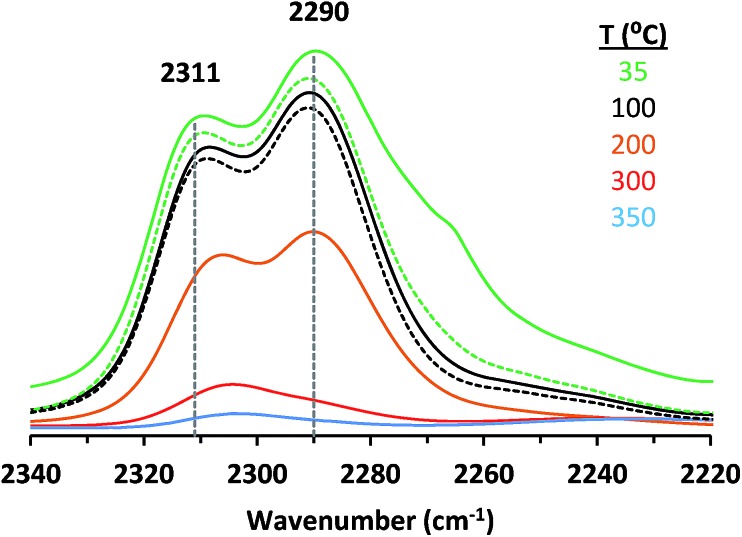
Baseline corrected IR spectra of CD_3_CN adsorbed on CIT-6 at 35 °C (green solid) and desorbed at different times and temperatures: 24 h at 35 °C (green, dashed), 0.5 h at 100 °C (black, solid), 1.5 h at 100 °C (black, dashed), 0.5 h at 200 °C (orange, solid), 0.5 h at 300 °C (red, solid), and 0.5 h at 350 °C (blue, solid).

**Scheme 1:**
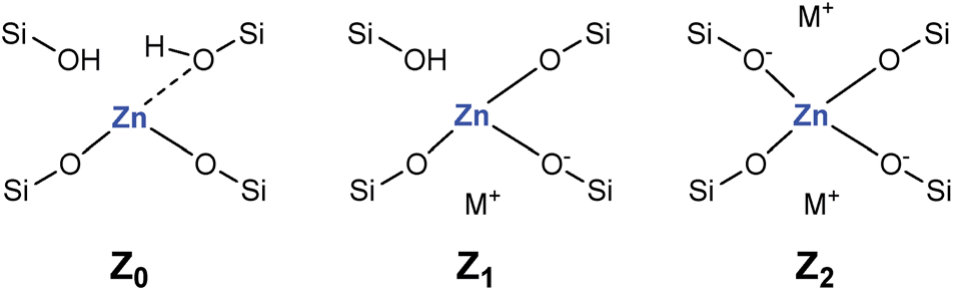
Proposed framework Zn site structures in microporous zincosilicates. M^+^ is a monovalent cation, such as alkali or alkyl ammonium.

Adsorption of CD_3_CN reveals the presence of at least two Lewis acid sites in CIT-6 (Fig. 2), with deconvoluted bands appearing at 2311 cm^–1^ and 2290 cm^–1^. The frequency of the 2311 cm^–1^ band of CIT-6 suggests an extent of polarization comparable to that generated by Sn-MCM-41, Sn-MFI, and Zr-beta (*ca.* 2309–2312 cm^–1^), but lower than that generated by the “open” Sn site of Sn-beta (2315 cm^–1^) or Al Lewis acid sites in various zeolites (≥2320 cm^–1^).^33^,^35^,^36^ This band is intermediate between the 2314 cm^–1^ band reported for the Zn-exchanged Al-beta^19^ and the 2305 cm^–1^ band measured for Zn sites dispersed on amorphous silica (Fig. S2†). The lower frequency of the band measured for the amorphous-supported Zn sites is consistent with the trend observed for Sn in Sn-beta and Sn-MCM-41.^36^,^37^ Silica-supported Zn sites have been previously characterized in the context of alkane dehydrogenation. EXAFS data suggest that the dehydrated Zn sites in this type of material datively coordinate an oxygen of a neighboring silanol (as shown in structure Z_0_ in Scheme 1),^22^ but this polarization appears insufficient to generate a strong Brønsted acid site that is capable of protonating pyridine.

The 2290 cm^–1^ band of CD_3_CN adsorbed on CIT-6 is likely associated with Li-bearing Zn sites. We previously observed a CD_3_CN band *ca.* 2292 cm^–1^ in a Sn-beta material that was exchanged with Li^+^ under basic conditions in order to cationate the neighboring silanol of the “open” Sn site.^21^ Similarly, Na^+^ and K^+^ exchanges generate sites with characteristic CD_3_CN bands *ca.* 2280 and 2273 cm^–1^, respectively. VPI-8 (VET framework) is a zincosilicate with a higher framework density than CIT-6 that can be found as a minor phase impurity in CIT-6 powders and is difficult to detect at low concentrations (as demonstrated by the data shown in Fig. S3†). VPI-8 crystallizes if the CIT-6 gel (which contains Li^+^ ions) is aged beyond complete CIT-6 crystallization^38^ and exhibits a CD_3_CN band primarily *ca.* 2294 cm^–1^. On the other hand, Zn-MCM-41 and Zn-MFI are synthesized from gels containing Na^+^ ions and CD_3_CN bands *ca.* 2280 cm^–1^ are observed for these materials. These data are consistent with the presence of Zn sites possessing structures Z_1_ and/or Z_2_ in Scheme 1. Further support for this tentative assignment comes from ion exchange experiments that shift the site distribution in CIT-6. A moderately basic Li^+^ exchange (1 M LiNO_3_, and initial pH = 10, set by LiOH) generates a material (CIT-6-LiEx) possessing primarily a 2290 cm^–1^ CD_3_CN band. On the other hand, exchanging CIT-6 with a nearly-neutral 1 M solution of N(CH_3_)_4_Cl, followed by calcination, produces a material (CIT-6-Z_0_) possessing primarily a 2312 cm^–1^ CD_3_CN band, with a broad red-shifted shoulder. It is important to note that, within the measurement error of energy dispersive spectroscopy (EDS), CIT-6-LiEx (Si/Zn = 10.9 ± 2.1) has the same Zn content as the parent material (Si/Zn = 12.2 ± 0.9), but CIT-6-Z_0_ loses nearly half of its Zn (Si/Zn = 21.3 ± 4.2). Materials with higher total Zn contents, with CD_3_CN bands *ca.* 2312–2310 cm^–1^, can be generated by an alternative post-synthetic strategy (*vide infra*). IR spectra of CD_3_CN adsorbed on VPI-8, Zn-MCM-41, Zn-MFI, and a number of post-synthetically Zn-modified materials are shown in Fig. S2† (powder XRD data of these microporous materials are included in Fig. S3†).

In our hands, CD_3_CN appears to interact much stronger with the two discernable Lewis acid sites in calcined CIT-6, than with any of the sites in Sn-, Ti-, or Zr-beta, or their alkali-exchanged counterparts. In fact, the persistence of coordinated CD_3_CN on the CIT-6 sites to temperatures beyond 200 °C under vacuum (Fig. 2) is consistent with the high temperatures of TPD desorption peaks for Zn-exchanged Al-beta.^19^ The high interaction strength of CD_3_CN with such sites (as inferred from high desorption temperatures) appears to conflict with the relatively low induced blue shifts of the CN vibration. This disparity may stem from the difference in the energies of structural rearrangements for Zn sites *vs.* Sn, Ti, or Zr sites upon desorption of probe molecules.

CD_3_CN adsorbed on bulk ZnO generates spectroscopic signatures distinct from CIT-6 (Fig. S4†), with a broad band *ca.* 2300 cm^–1^ that can be attributed to Lewis or Brønsted acid sites, as well as a multitude of bands below 2200 cm^–1^, characteristic of CD_2_CN^–^ and polyanions formed by deprotonation of CD_3_CN by strongly basic surface oxygen species.^34^ While the 2300 cm^–1^ band is not spectroscopically resolved from the bands observed in CIT-6, the lack of CD_2_CN^–^ signatures in the CIT-6 spectra suggests absence of detectable amounts of extra-framework ZnO in CIT-6 after calcination.

### 3.2. Catalysis with microporous zincosilicates

**Fig. 3:**
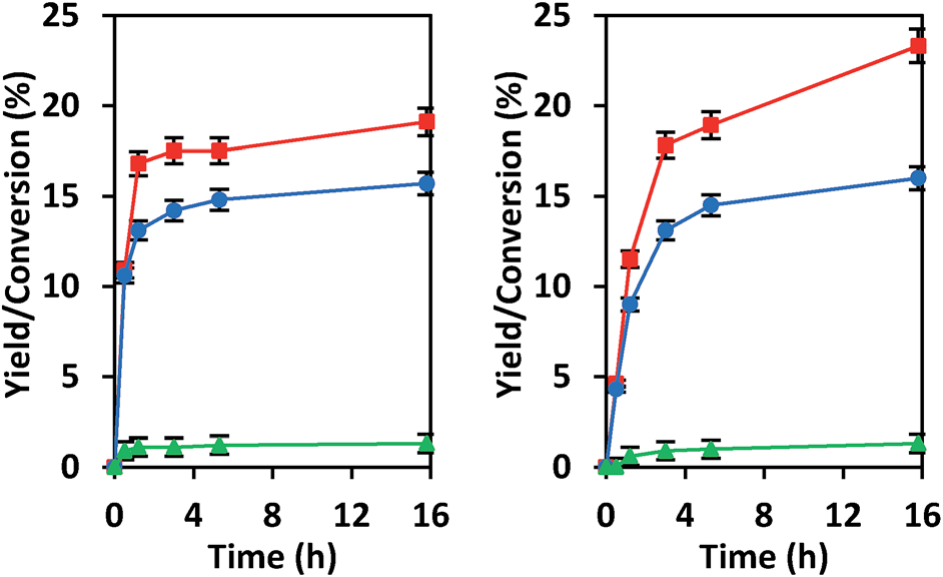
Glucose isomerization reactions are catalyzed by CIT-6 in water (left) and methanol (right) solvents. Glucose conversion (red squares), fructose yields (blue circles), and mannose yields (green triangles) are plotted as a function of reaction time. Reaction conditions: 100 °C, 1% (wt/wt) glucose, 1 : 50 Zn : glucose initial molar ratio.

We have previously explored reactions of sugars catalyzed by isolated Sn sites in SiO_2_-based materials and by SnO_*x*_ particles located in the pores of Si-beta.^39^ The isolated “open” Sn sites that have an adjacent protonated silanol were found to promote glucose–fructose isomerization through a 1,2-intramolecular hydride shift mechanism, while SnO_*x*_ particles behaved as base catalysts by promoting the same isomerization through an enolate mechanism that involves deprotonation of α-carbonyl carbon (Scheme S1†).^21^,^39^ Data provided in Fig. 3 show that under the same reaction conditions as we used previously, CIT-6 isomerizes glucose to fructose in both water and methanol solvents, but does so with proton abstraction from the α-carbonyl carbon (as evidenced by ^13^C NMR of isotopically labeled glucose; Fig. S5†). While the reaction appears to proceed catalytically (TOF > 1 in water and methanol), the reaction slows prior to reaching an equilibrium distribution of sugars, suggesting that catalyst deactivation occurs (Fig. S6†).

EDS analysis indicates a 35% decrease in Zn content of the CIT-6 sample after reaction in water. Recalcination of the catalyst recovered and washed after such a reaction does not result in the recovery of isomerization activity. Additionally, CD_3_CN adsorption on this material reveals a loss of the 2290 cm^–1^ band that is present in the original CIT-6 sample, but retention of the 2311 cm^–1^ band (Fig. S7†). Though ZnO and Zn(OH)_2_ can catalyze the isomerization glucose through the enolate pathway, it is unlikely that the isomerization activity observed in the original CIT-6 is attributable to such species, as their presence is not observed in spectroscopic characterization, and the permanent catalyst deactivation is inconsistent with their presence. The catalytic data obtained from CIT-6 are more consistent with involvement of Z_1_ or Z_2_ sites that we hypothesize are correlated to the 2290 cm^–1^ CD_3_CN band. The enhanced basicity of framework oxygens whose charge is balanced by alkali cations may allow for participation of Z_1_ or Z_2_ sites in this base-promoted reaction. The generation of small quantities of α-hydroxycarboxylic acid species (*e.g.*, lactic acid), as ascertained by ^1^H and ^13^C NMR, suggests acid-catalyzed hydrolysis of Z_1_ and Z_2_ sites as the possible mode of deactivation.

**Fig. 4:**
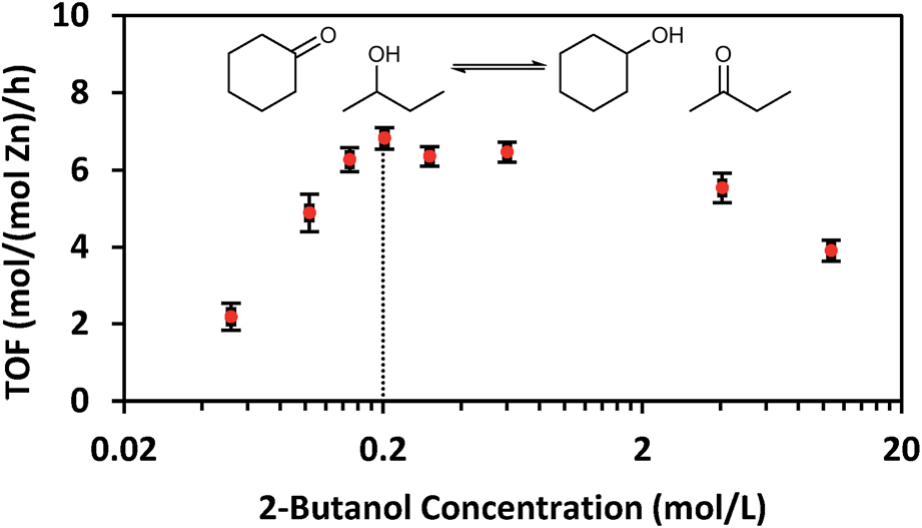
Initial TOF of MPVO reactions of cyclohexanone and 2-butanol catalyzed by CIT-6 as a function of 2-butanol concentration. Reaction conditions: 100 °C, 0.2 M cyclohexanone, indicated concentration of 2-butanol in cyclohexane, and 1 : 100 Zn : cyclohexanone initial molar ratio.

While CIT-6 is an active catalyst for the glucose–fructose isomerization, this reaction is not proceeding *via* catalysis by the Lewis acid sites (as probed by base adsorption and IR). The p*K*_a_ values of the conjugate acids of nitriles are higher than those of the conjugate acids of aldehydes, ketones, alcohols, and water. Outside of solvation effects, this relative ranking implies that nitriles should have weaker interactions with Lewis acid sites than the other Lewis basic species listed above. Data provide in Fig. 2 show that vacuum desorption of CD_3_CN from CIT-6 Lewis acid sites at 100 °C is slow, with minimal desorption occurring over the course of an hour. Desorption of water, methanol, and cyclohexanone were also observed to be slow at 100 °C, and appreciable desorption rates only occurred at temperatures higher than 200 °C. These low rates of desorption of probe molecules are expected to translate to slow desorption of these types of compounds present in solutions at reaction conditions. Thus, the Lewis acid-mediated catalytic properties of Zn sites in CIT-6 at low temperatures are mitigated in solvents possessing strongly Lewis basic functional groups. This interpretation is supported by the observed rate behavior of Lewis-acid mediated MPVO reactions of cyclohexanone and 2-butanol catalyzed by CIT-6. Data in Fig. 4 show the measured initial rates for this reaction as a function of 2-butanol concentration, for a fixed concentration of cyclohexanone (0.2 M). The initial rate of the reaction increases with increasing 2-butanol concentration, but peaks at a concentration where the ratio of the two reactants is stoichiometric (0.2 M), and decreases with further increase in 2-butanol concentration. This reaction behavior is consistent with kinetically limiting desorption rates. The nominal TOF of CIT-6 for this reaction (based on total Zn) is 3–4 orders of magnitude lower than that of Sn-beta (based on total Sn) under similar reaction conditions.^3^ At this point, it is not clear if the low TOF reflects the intrinsic catalytic activity of the Zn sites and their hindered desorption rates, or if severe diffusion limitations induced by high concentrations of strongly bound species also contribute to the slow measured kinetics.

Sabatier's principle can be used to qualitatively rationalize the poor catalytic performance observed for MPVO reactions promoted by CIT-6 relative to the performance of other Lewis acidic beta zeotypes known to catalyze such reactions,^2^,^40^ and to infer reaction conditions where CIT-6 may behave as a catalytically interesting material. The principle states that for a reaction with a given activation energy, an optimal enthalpy of desorption of the substrate from the heterogeneous catalyst exists. For MPVO reactions, Ti-beta appears to interact too weakly and CIT-6 too strongly with the carbonyl bearing substrates (as inferred from desorption of cyclohexanone), with both cases resulting in low reaction rates. Sn-beta and Zr-beta have desorption rates intermediate to the two extremes and result in significantly higher reaction rates at a given temperature. Because these materials usually possess more than one kind of coordination environment for the heteroatoms, quantitative statements of these results is presently not possible due to a lack of measurements of site-specific reaction rates and adsorption enthalpies. Another outcome of Sabatier's principle suggests that reactions with higher intrinsic activation barriers will generally correspond to higher optimal adsorption enthalpies. Thus, the Lewis acid sites of CIT-6 have the potential to catalyze high-temperature reactions more optimally than the Lewis acid sites in weaker binding materials such as in Ti-, Zr-, and Sn-beta.

**Scheme 2:**
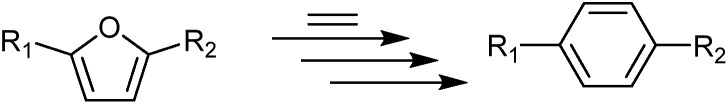
Generalized description of Diels–Alder cycloaddition–dehydration reactions of substituted furans (R_1_, R_2_ = CH_3_, CH_2_OR, CHO, or CHOOR, where R = H or alkyl group). This is a multistep process, whose rate limiting steps are greatly influenced by the identity of the R_1_ and R_2_ groups.

The Diels–Alder (DA) cycloaddition–dehydration reactions of substituted furans with ethylene are a promising route to terephthalic acid made from renewable sources (Scheme 2). Such reactions of dimethyl furan (R_1_ = R_2_ = CH_3_ in Scheme 2) can be catalyzed more efficiently by Brønsted acids than Lewis acids,^41^,^42^ but furans with oxygenated side-groups that can be derived from 5-hydroxymethylfurfural without costly reduction steps react on Brønsted acidic zeolites with negligible selectivities towards desired DA products under similar or milder conditions.^13^ Only the Lewis-acidic, Sn- and Zr-beta were previously observed to catalyze the DA reactions of such substrates with appreciable selectivity, with Zr-beta resulting in considerably higher selectivities, for reasons currently not understood.^13^ The apparent absence of strong Brønsted acid sites in CIT-6 makes it a candidate catalyst for these kinds of DA reactions.

**Fig. 5:**
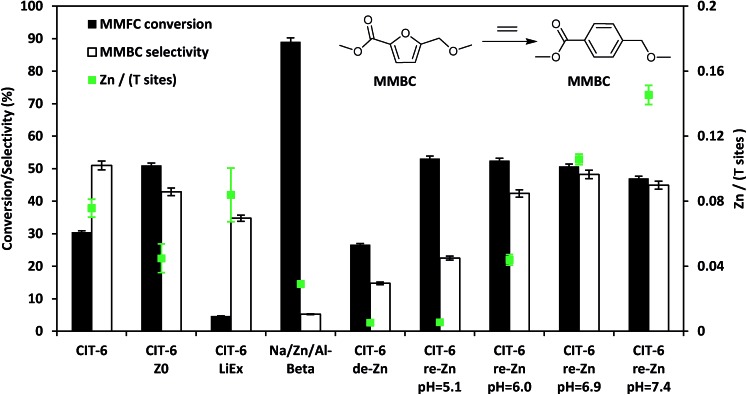
Diels–Alder cycloaddition–dehydration reactions of MMFC with ethylene catalyzed by CIT-6 and its various modified forms with different Zn contents and site distributions.

In heptane solvent, CIT-6 catalyzes the formation of methyl 4-(methoxymethyl) benzenecarboxylate (MMBC) in the reaction of methyl 5-(methoxymethyl)furan-2-carboxylate (MMFC) with ethylene at 190 °C, with a yield of 16% at 51% selectivity (Fig. 5). No significant quantities of soluble byproducts were detected by ^1^H NMR (Fig. S8†) or observed in the GC chromatograms. Interestingly, no conversion is observed in dioxane, the solvent found to give the best selectivity for this reaction when Zr-beta or Sn-beta catalysts are used.^13^ This result is consistent with the hypothesis that, in CIT-6, there is Lewis acid site passivation through competitive binding of oxygenated species, even at these relatively high temperatures. Furthermore, Brønsted acid sites are expected to not be passivated in dioxane, and the lack of MMFC conversion in this solvent suggests that no catalytically relevant Brønsted acid sites are accessible at the reaction temperatures. Additionally, bulk ZnO produces no detectable conversion of MMFC in heptane, demonstrating that it is the framework Zn in CIT-6 that is catalytically active in this reaction. CIT-6-LiEx is significantly less active than the parent CIT-6, suggesting that the CIT-6 sites associated with 2290 cm^–1^ CD_3_CN IR band are unable to effectively catalyze these reactions. CIT-6-Z_0_ primarily has sites associated with the 2311 cm^–1^ CD_3_CN IR band and, despite its lower Zn content, results in higher MMBC yields than the parent CIT-6 material. These data implicate the Z_0_ sites as the catalytically active species in such DA cycloaddition–dehydration reactions. However, site cooperativity in mixed-site samples of CIT-6 is another possibility that warrants consideration in future studies. Because determination of cooperativity would require quantitation of each type of site, determination of their proximity, and measurement of intrinsic site kinetics, such efforts are outside of the scope of this work.

While other porous zincosilicate materials (Zn-MCM-41, Zn-MFI, VPI-8, and deboronated SSZ-33 that has been post-synthetically zincated) have varying distributions of Zn Lewis acid sites (*i.e.*, different proportions of Z_0_, Z_1_, and Z_2_ type of sites as characterized by CD_3_CN IR experiments (Fig. S2†)), all of these materials produce negligible levels of MMBC and result in minor conversion of MMFC and appear brown in color after reaction (Table S1†). The reason for the lack of activity of Zn sites in these materials remains unknown. However, the 10MR pores in Zn-MFI may be too small. VPI-8 (VET structure) has a 1-D linear 12MR pore system and SSZ-33 (CON structure) has 12MR pores intersected by 10 MR pores, so both materials should be able to accommodate molecules that can enter the *BEA framework, but the additional limitation of 1-D diffusion may render these materials effectively inactive. The lack of activity of the un-constrained sites in Zn-MCM-41 or Zn on amorphous silica are hard to reconcile, but these results are consistent with the reported lack of activity of Sn- and Zr-MCM-41,^13^ and suggest that the *BEA framework may play a role in the stabilization of intermediates or transition states for such DA reactions. We also note that Na–Al-beta that has been ion exchanged with Zn^2+^ leads to negligible selectivity towards MMBC, and apparent coking of catalyst.

Assuming homogeneous dispersion of Zn in CIT-6, the relatively low Si/Zn ratio of the material (12.2 ± 0.9) implies that 4.8 Zn sites are found per unit cell. Considering that each MMFC molecule possesses 4 oxygen atoms, and spans a large fraction of the void space of a *BEA unit cell, coordination to multiple Zn sites is possible at high Zn loadings. Multiple coordination points with the framework are expected to further increase the molar enthalpy of desorption for reactants, intermediates, and products. The close spacing of Zn sites also increases the likelihood of coupling reactions, as adsorbates may be positioned in intimate contact for extended periods of time. We investigated the possibility of increasing catalyst selectivity by adapting a procedure used to ion-exchange Zn amines onto amorphous silica supports in order to generate dispersed Zn sites primarily in Z_0_ configuration, as characterized by CD_3_CN IR experiments (Fig. S2†).^24^ Briefly, this procedure involves contacting a material possessing silanol nests with an aqueous solution containing 0.1 M ZnCl_2_, and 2.0 M NH_4_Cl, adjusted to desired pH by NH_4_OH or HCl, followed by recovery and washing of solids, drying, and calcination. For amorphous silica, this procedure is reported to generate materials with Si/Zn ratios as low as 9 when the pH of the starting solution is 7.4, while decreasing pH of the starting solution leads to lower Zn contents.^24^ CIT-6 exposed to 1.0 M H_2_SO_4_ for 12 h, at ambient temperatures, (denoted CIT-6-de-Zn) loses most of its Zn (Si/Zn = 188 ± 17 is near the detection limit of EDS), leaving behind silanol nests that can host re-introduced Zn. Indeed, the application of the Zn–amine-based procedure leads to incorporation of Zn in CIT-6-de-Zn, with higher pH corresponding to higher Zn incorporation and higher MMBC yields (up to pH = 6.9), at nearly identical conversions (see entries in Fig. 5 denoted as CIT-6-re-Zn-pH = *X*, where *X* corresponds to the initial pH in the re-zincation solution). At Si/Zn = 5.8 ± 0.2, CIT-6-re-Zn-pH = 7.4 has a nominal Zn loading that exceeds that of the original material by a factor of 2. Such high Zn loading either suggests that the original CIT-6 material, prior to de-zincation, already possesses a number of unoccupied silanol nests, or that immobilization of extra-framework Zn species may also occur at higher pH.

Interestingly, CIT-6-de-Zn converts a significant amount of MMFC (27%), with low selectivity towards MMBC (15%). These data suggest that the side reactions do not correlate with decreased Zn spacing, but reveal that underlying non-selective sites may exist in CIT-6. Standard CIT-6 is synthesized in hydroxide media with Ludox AS-40 as the silica source. Thus, the byproduct-forming sites may be either highly active impurities from Ludox (Al or Fe) that are not removed by the acid treatment, or the numerous silanol defects that are generated upon de-zincation.

While the MMBC selectivity of CIT-6, or the various post-synthetically generated Zn-containing beta zeotypes presented here, is still lower in heptane than that reported for Zr-beta in dioxane (∼70–80%), it is higher than that of Sn-beta in dioxane (∼50%).^13^,^43^ Furthermore, a number of reactions reported here exceed the selectivity of Zr-beta in heptane under identical reaction conditions (measured to be 42% here), and result in comparable net yields of MMBC. The MMBC selectivity of CIT-6 can be further increased to 62% by lowering the reaction temperature to 170 °C (Table S1†).

Another interesting and more stable furan, 2,5-furandicarboxylic acid (FDCA), is formed upon oxidation of both side-groups of 5-HMF to carboxylic acid groups, and can be obtained in high yield and selectivity from 5-HMF, or can be made from fructose, with 5-HMF as an intermediate in a “one-pot” scheme.^44^,^45^ The dimethyl ester of FDCA, dimethyl 2,5-furandicarboxylate (DMFDC), is used in its purification by vacuum distillation, and can be formed either through a separate esterification of FDCA, or directly and quantitatively during the oxidation of 5-HMF.^46^ DA cycloaddition–dehydration reactions of ethylene with FDCA and DMFDC (R_1_ = R_2_ = COOH or COOMe in Scheme 2, respectively), are attractive direct routes to terephthalic acid (TPA) and dimethyl terephthalate (DMT), respectively, that do not require subsequent oxidation steps. Prior investigations of such reactions catalyzed by Sn-beta showed that both of these substrates were highly resistant to DA reactions. Only under forcing conditions (300 °C) did DMT form from DMFDC in dioxane solvent, with a molar yield of 0.4% and >1% selectivity.^13^,^47^ A BP patent reports no formation DMT, but a molar yield of 0.023% of TPA, at 0.038% selectivity, when DMFDC is reacted with ethylene without a catalyst in toluene solvent at 190–195 °C.^48^ Similarly a Furanix – Coca-Cola patent application reports a 7.2–11.6% yield of TPA, at an undisclosed conversion or selectivity, without any DMT observed, when DMFDC is reacted with ethylene in homogeneous acid/acetic anhydride mixtures at 240 °C.^49^

**Fig. 6:**
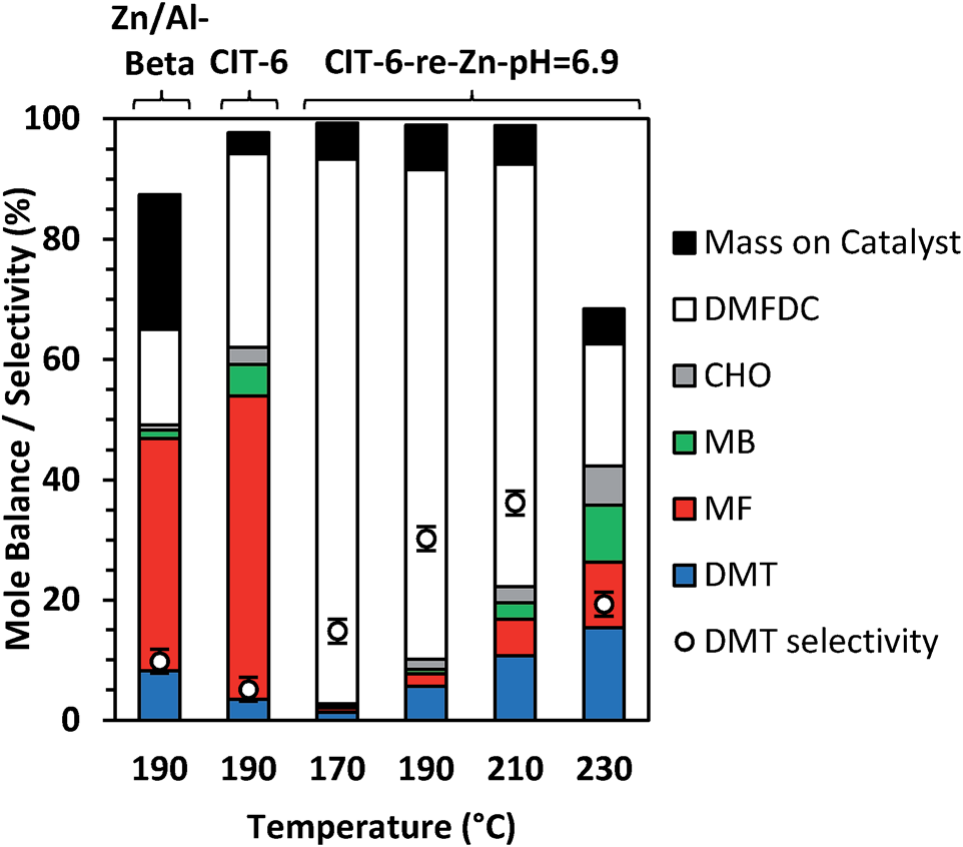
Diels–Alder cycloaddition–dehydration reactions of DMFDC catalyzed by CIT-6 and CIT-6-re-Zn-pH = 6.9 at various temperatures. Resulting yields (%) of DMT, MF, MB, CHO, and DMFDC are calculated as ratio of moles formed to initial moles of DMFDC. Mass on catalyst (%) is expressed as ratio of combustible mass on catalyst (measured by TGA) to initial mass of DMFDC. Reaction conditions: 12 h, effective concentration of 0.033 M DMFDC in 10 mL heptane, 100 mg of catalyst, and 35 bar C_2_H_2_ at 25 °C.

**Scheme 3:**
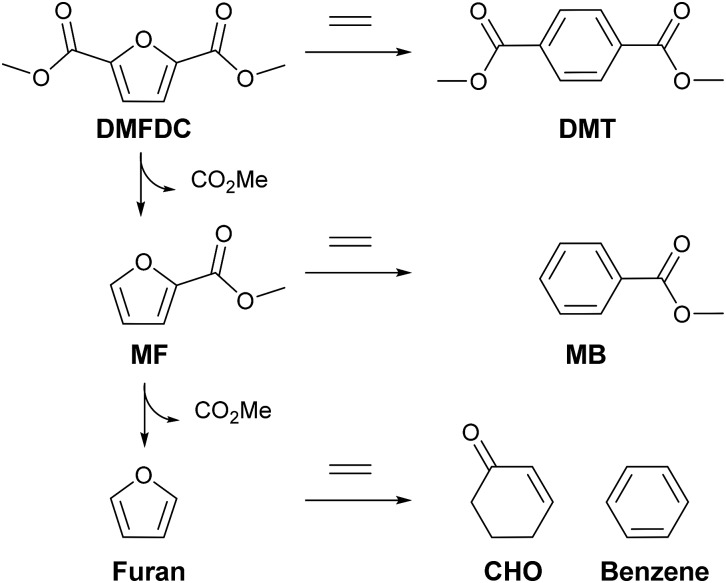
Full Diels–Alder cycloaddition–dehydration reaction diagram for DMFDC as a substrate. In the case of CIT-6, decarboxylation reactions are primarily catalyzed by Li-bearing sites.

CIT-6 is able to catalyze the formation of DMT from DMFDC at 190 °C, with a yield of 3.4% and 5.1% selectivity (Fig. 6). GC-MS and ^1^H NMR spectra (Fig. S9 and S10†) of the reaction solutions indicate significant quantities of three byproducts: methyl 2-furoate (MF), methyl benzoate (MB), and 2-cyclohexenone (CHO), at 50.5%, 5.3%, and 2.8% yield (measured by GC-FID), respectively. MF is hypothesized to form through decarboxylation reactions of DMFDC, while MB may form either through a DA step with ethylene from MF, or as a decarboxylation product from DMT. No significant formation of MB was observed when DMT was used as a reactant, suggesting that the latter scenario is not likely. CHO is potentially formed from the DA product of ethylene and furan, as shown in Scheme S2.† After the DA cycloaddition, the resulting 7-oxabicyclo[2.2.1]hept-2-ene adduct can rearrange to an epoxide, as is proposed to occur in the Lewis acid catalyzed dehydration of the DA adduct of dimethyl furan.^42^ Subsequently, the epoxide can be isomerized into the enone by a Lewis-acid-promoted hydride shift.^50^ The presence of this product suggests that furan and benzene should also form, but these species are only observed in GC-MS at very small relative amounts, and are poorly resolved from solvent peaks. Furthermore, because these species are significantly more volatile than any other component of the reaction solution they may be removed in part during vessel depressurization. For these reasons, we did not attempt to quantify the yields of furan and benzene in this work. Scheme 3 summarizes the hypothesized reaction network.

With Sn-beta catalysts, the disodium salt of FDCA was reported to primarily decarboxylate rather than produce DA products.^47^ Furthermore, decarboxylation reactions of esters are generally hypothesized to proceed through free acid or carboxylate intermediates, which may be stabilized by alkali ions.^51^ Thus, the Li-bearing sites of CIT-6 were suspected as a potential source for such decarboxylation reactions. CIT-6-re-Zn-pH = 6.9 does not possess Li-bearing sites, and results in nearly double DMT yield (5.6%) and significantly higher selectivity (30.2%) than the parent CIT-6 material. Furthermore, elimination of the Li-bearing sites significantly reduces the production of MF, MB, and CHO to 2.1%, 0.8%, 1.7%. Analogous decarboxylation products, 2-(methoxymethyl)furan and (methoxymethyl)benzene, were not observed in product solutions of MMFC reactions with Li-containing catalysts (Fig. S8†), but such species may be intermediates in coking that is observed for the MMFC reactions on CIT-6. While undesirable here, sites responsible for decarboxylation reactions of FDCA may be of interest in the context of Henkel reactions that have been shown to be catalyzed by ZnCl_2_.^52^ We note that at 190 °C, Zn/Al-beta also catalyzes the formation of DMT, with a 9.0% DMT yield and 10.8% selectivity. Significant amounts of decarboxylation products are observed with this material, with 36.4%, 1.4%, and 0.7% yields of MF, MB, and CHO, respectively.

TGA mass losses above 250 °C for the acetone-washed catalyst from DMFDC reactions catalyzed by CIT-6 and CIT-6-re-Zn-pH = 6.9 correspond to 3.4% and 7.4% of the initial DMFDC mass, respectively (after adjusting for any solvent retention, determined by TGA of acetone-washed, unreacted catalyst). Thus, despite the large difference in conversion and side-product formation, the difference in substrate adsorption or coking is minor. Additionally, unlike the yellow-brown color of catalysts after reactions of MMFC, both catalysts appear white after DMFDC reactions. In contrast, the Zn/Al-beta catalyst turned red-brown after DMFDC reactions, suggesting a greater extent of coking occurred on this catalysts, as corroborated by the increase in TGA mass loss to 22.5% of initial DMFDC mass. Mole balance data (based on moles DMFDC) for CIT-6 and CIT-6-re-Zn-pH = 6.9, for temperatures ranging from 170 °C to 230 °C, are shown in Fig. 6. The yield of DMT progressively increases with temperature, and despite the accompanying growth of MF, MB, and CHO, the apparent selectivity for DMT also increases up to 36.2%, at 210 °C, as the combustible mass deposited onto the catalyst does not appear to change significantly, and constitutes a progressively smaller fraction of the conversion. The large decrease in mole balance closure at 230 °C is not accompanied by increase in coking or detectable formation of new product species (as characterized by GC and NMR); however, volatile species like benzene and furan escape quantification under the current experimental protocols, and may constitute a larger fraction of the conversion.

Preliminary investigation of catalyst stability and recyclability were performed with CIT-6-reZn-pH = 6.9 for the DA reaction of DMFDC and ethylene at 210 °C. These experiments suggest that, while the catalyst retains activity without intermediate calcination, the product distribution after each run changes, with decarboxylation reactions becoming more prominent, and contributing to decreased DMT selectivity (Fig. S11†). Furthermore, calcination of the catalyst after the second reuse does not restore the initial selectivity. Neither the XRD pattern of (Fig. S12†) nor the qualitative IR spectrum of CD_3_CN adsorbed on (Fig. S13†) the catalyst recovered after the third run are appreciably changed. Within certainty of EDS measurements, the Si/Zn ratio of the catalyst also remained constant throughout these experiments (Fig. S14†). Thus, more extensive recyclability tests and new probes for the changes to active site structure are needed to understand the evolution of the catalyst activity and selectivity.

The net processes for biomass-based production of DMT involve the synthesis and purification steps of the furan reactants, as well as any subsequent product processing. DMFDC is a more attractive substrate from the standpoint of its stability and the elimination of oxidation steps after the DA reactions. The data presented here show that isolated Zn sites in the *BEA framework offer significant improvement in both yield and selectivity of DA cycloaddition–dehydration reactions of ethylene and DMFDC over previously investigated heterogeneous catalysts. While both metrics remain lower for the DA reactions of DMFDC than for MMFC, these results indicate that such reactions are feasible, and warrant further consideration. Additionally, the identified side products of DMFDC reactions, MF, MB, CHO, furan, and benzene, have industrial applications, and do not correspond to complete loss of carbon.^53^,^54^

## 4. Conclusions

Framework Zn sites in microporous zincosilicates behave as Lewis acid centers in probe-molecule IR spectroscopy, with unusually high adsorption energies of Lewis bases on such materials. The strong interactions with adsorbates severely limit the activity of zincosilicates at low temperatures and in solvents bearing Lewis basic groups for reactions catalyzed by Lewis acids (*e.g.* inter- and intra-molecular MVPO reactions). However, at higher temperatures, in heptane solvent, CIT-6 (Zn-beta) is able to catalyze Diels–Alder cycloaddition–dehydration reactions of ethylene with methyl 5-(methoxymethyl)furan-2-carboxylate, a promising route to biomass-based terephthalic acid. Furthermore, a CIT-6-based catalyst enables the use of the dimethyl ester of furan-2,5-dicarboxilic acid, a furan resistant to Diels–Alder cycloaddition–dehydration reactions catalyzed by known Lewis acid zeotypes (*e.g.*, Sn-beta), resulting in direct formation of dimethyl terephthalate without the need for further oxidation reactions. Elimination of alkali-bearing sites is shown to significantly improve the selectivity of such reactions towards dimethyl terephthalate by lowering the extent of decarboxylation side-reactions that result in the formation of the notable byproducts: methyl 2-furoate, methyl benzoate, and 2-cyclohexenone. Here, only zincosilicates with *BEA topology have been demonstrated to be of catalytic interest, but probe-molecule IR characterization suggests that the Lewis-acidic character of isolated Zn sites in pure-silica frameworks is general, and that such materials warrant broader consideration for high temperature catalytic applications, especially those sensitive to the presence of strong Brønsted acid sites.

## Supplementary Material

Supplementary information
