# Supplementary material for: Catalysis by framework zinc in silica-based molecular sieves
Source: Chem Sci. 2016 Jan 4;7(3):2264–74. doi: 10.1039/c5sc03889h (PMC5977500; doi:10.1039/c5sc03889h)
Supplement: Supplementary file 1 [file SC-007-C5SC03889H-s001.pdf]

Electronic Supplementary Material (ESI) for Chemical Science.
This journal is © The Royal Society of Chemistry 2016

Catalysis by framework zinc in silica-based molecular sieves

Supporting Information

1. Spectroscopic and catalytic characterization of microporous zincosilicates

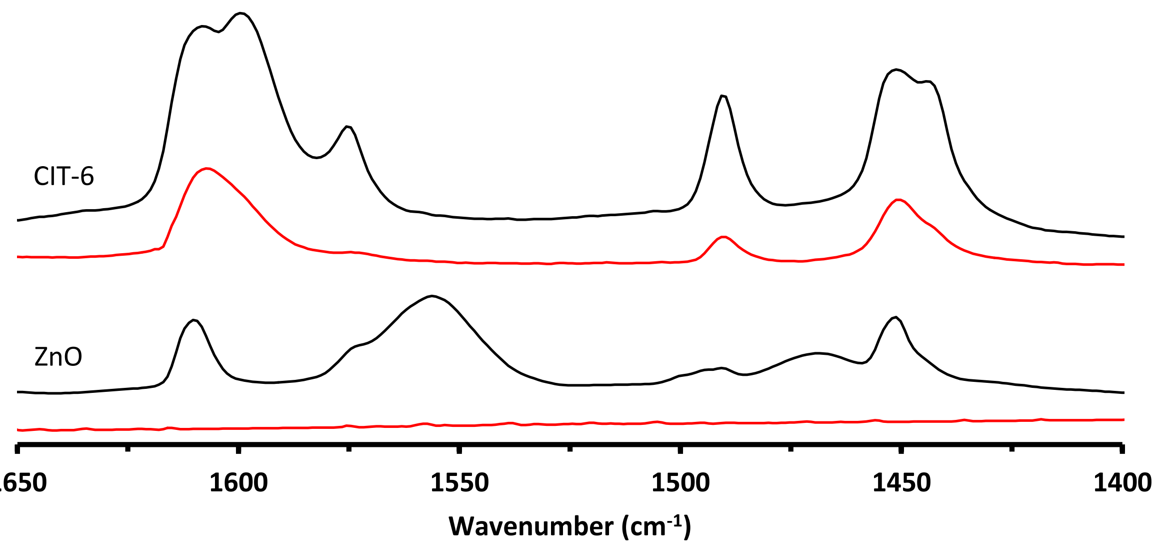

Fig. S1: Baseline-corrected IR spectra of pyridine adsorbed on CIT-6 (top) and ZnO (bottom) at 35 °C. Black and red spectra correspond to subsequent desorption under dynamic vacuum for 1h at 100 °C and 300 °C, respectively.

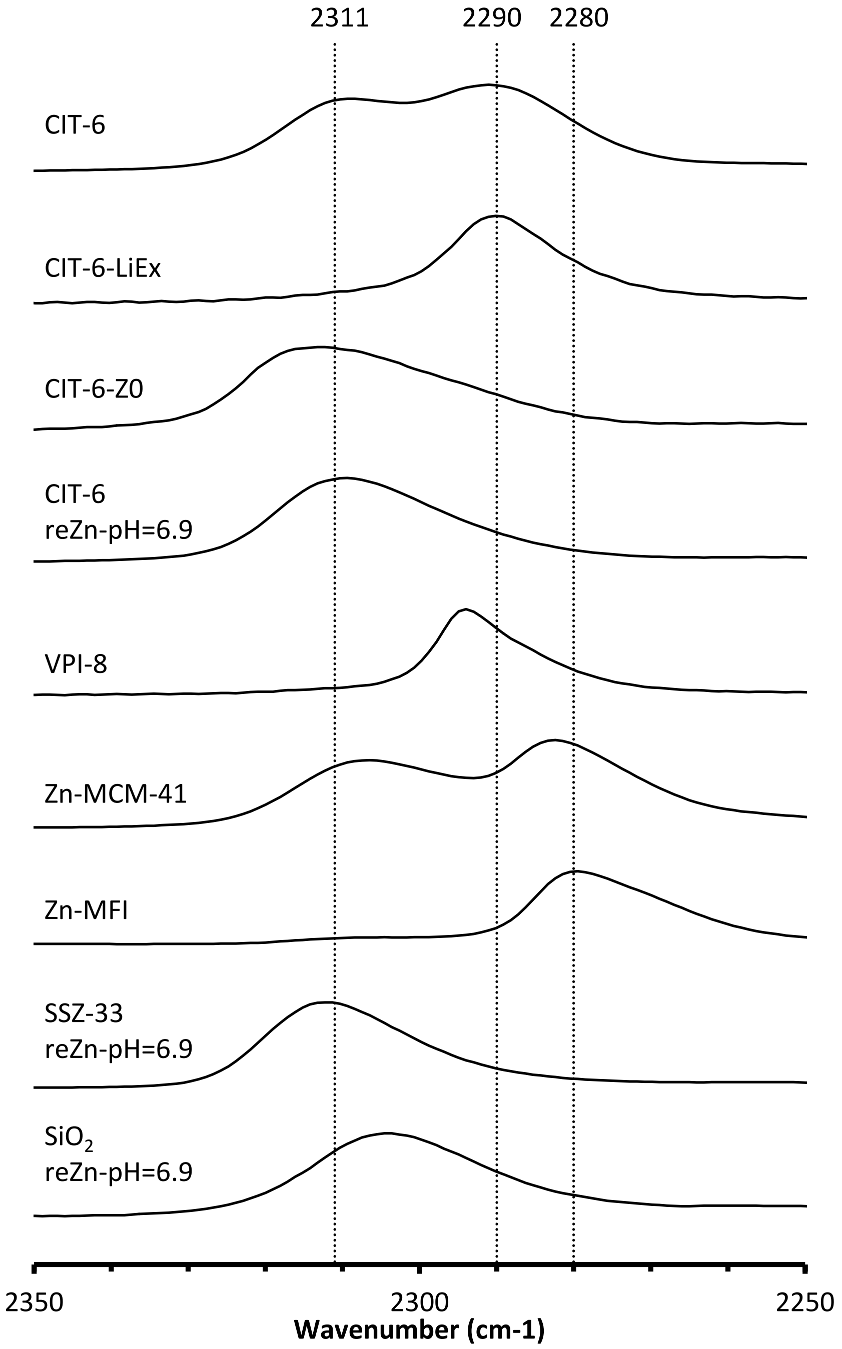

Fig. S2: Baseline-corrected, normalized IR spectra of CD_3CN adsorbed at 35 °C on (from top to bottom): CIT-6, CIT-6-LiEx, CIT6-Z0, CIT-6-reZn-pH=6.9, VPI-8, Zn-MCM-41, Zn-MFI, SSZ-33-reZn-pH=6.9, and SiO_2-reZn-pH=6.9. Spectra were collected after desorption at 100 °C for 1h.

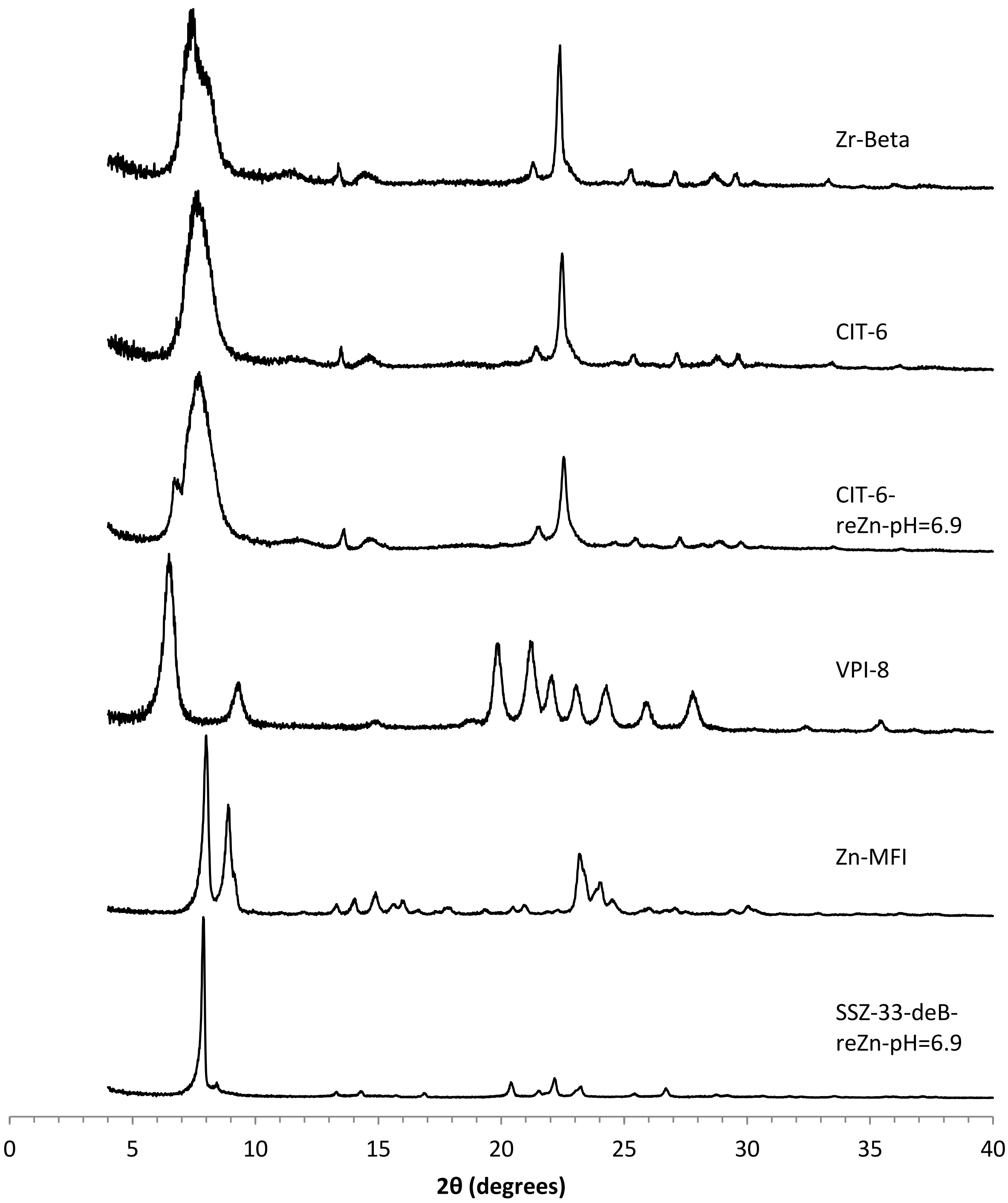

Fig. S3: Normalized powder XRD data for selected microporous materials (from top to bottom): Zr-Beta, CIT-6, CIT-6-reZn-pH=6.9, VPI-8, Zn-MFI, and SSZ-33-reZn-pH=6.9. All materials have been calcined. XRD pattern of the parent CIT-6 sample does not have noticeable VPI-8 peaks, but, upon 1M H_2SO_4 treatment, Zn-reinsertion, and calcination, a shoulder in the low angle peak of *BEA becomes apparent, indicating the presence of VPI-8 (VET framework) as a minor phase impurity. Crystal aggregates of VPI-8 morphology are also observed among *BEA crystals. The increase in the prominence of the VET peak in the powder pattern may be associated with selective partial degradation of the *BEA framework in the treatment of the sample, as it is a less dense structure.

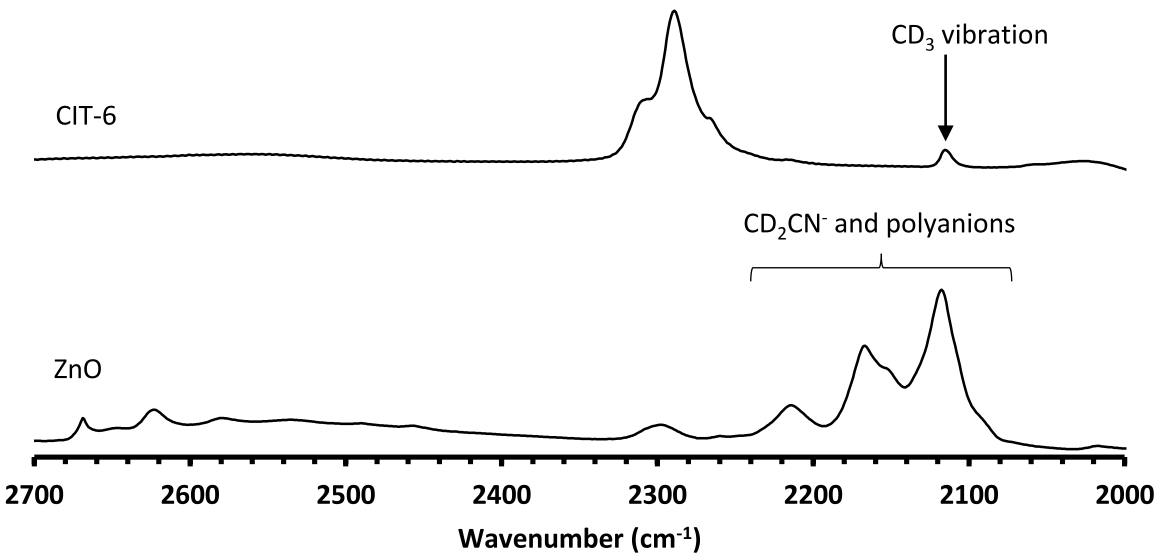

Fig. S4: Baseline-corrected, normalized IR spectra of CD_3CN adsorbed at 35 °C on CIT-6 (top) and ZnO (bottom). A CD_3 vibration band ca. 2115 cm^{-1} is found in all spectra of CD_3CN adsorbed on SiO_2 materials, and is not a shifted CN band. Spectra were collected after desorption at 35 °C for 1h.

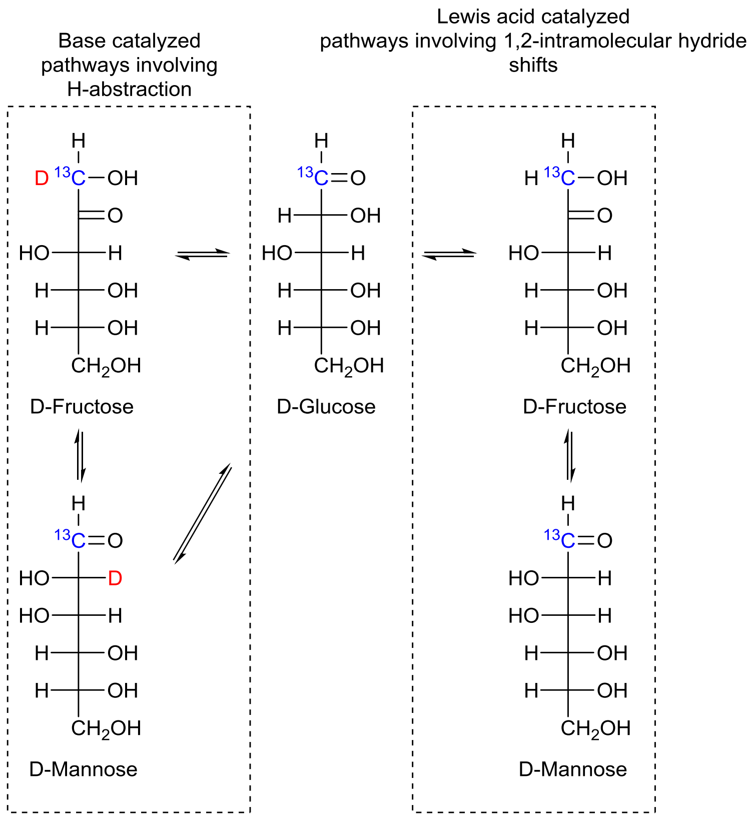

Scheme S1: Illustration of glucose isomerization mechanisms promoted by bases and Lewis acids. For reactions performed in deuterated solvents (D_2O or MeOD), deuterium incorporation is expected for products formed through enolate intermediates, but not through intramolecular hydride shifts. The use of ^{13}C-C1-glucose enables product analysis without the need for fractionation.

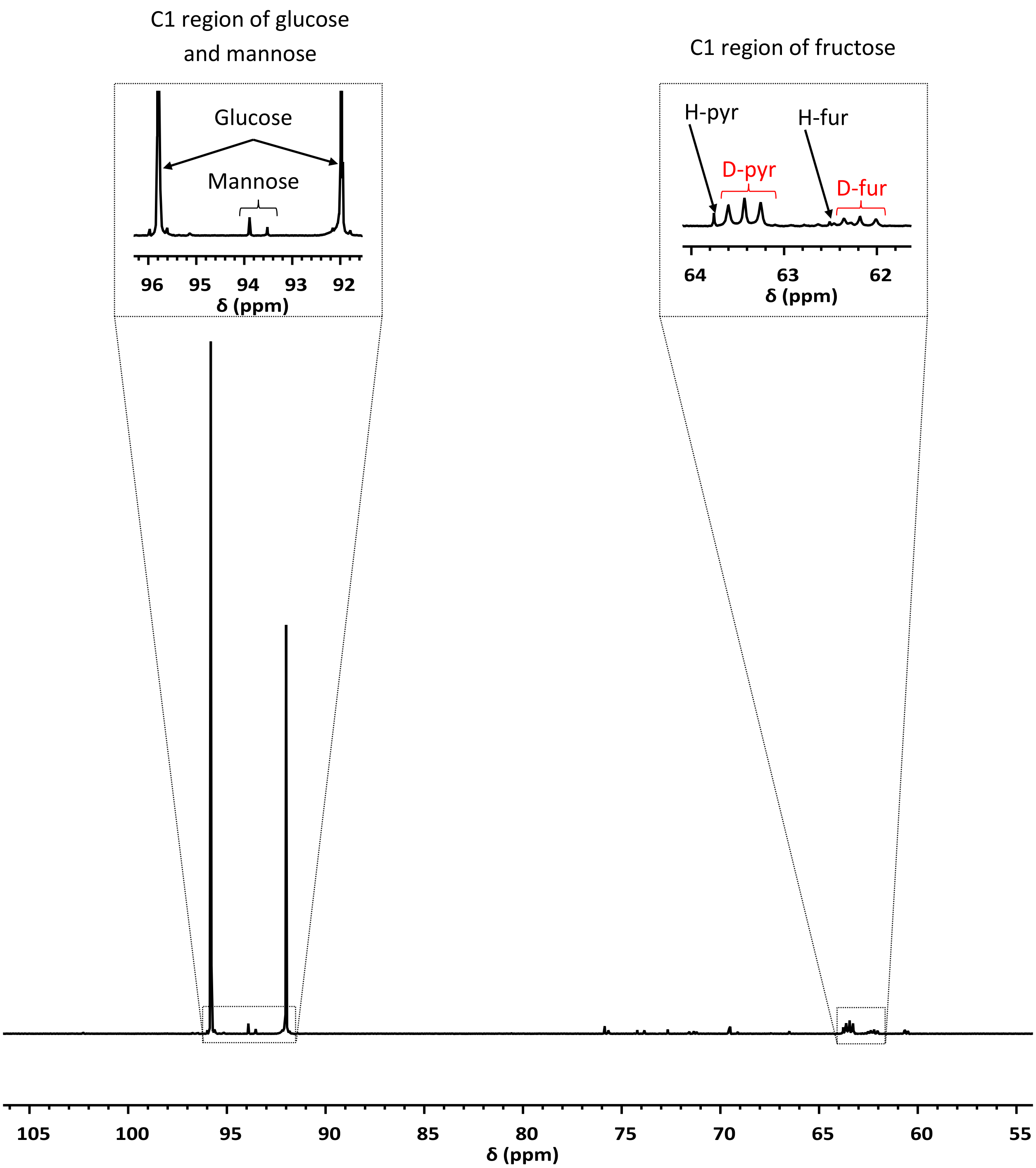

Fig. S5: ^{13}C NMR spectrum of unseparated reactant (^{13}C-C1-glucose) and the products generated by CIT-6 at 100 °C after a 1h reaction in D_2O. The abbreviations "pyr" and "fur" stand for pyranose and furanose, respectively. Incorporation of deuterium at C1 position of fructose, as evidence by appearance of low-intensity triplets, indicates a base-catalyzed mechanism. The presence of ^{1}H-form of fructose likely originates from the small fraction of ^{1}H impurity in D_2O solvent, but could also arise from a small contribution from a hydride shift mechanism. Unlabeled peaks correspond to natural abundance ^{13}C (~1%) occurring in glucose C2-C6 positions.

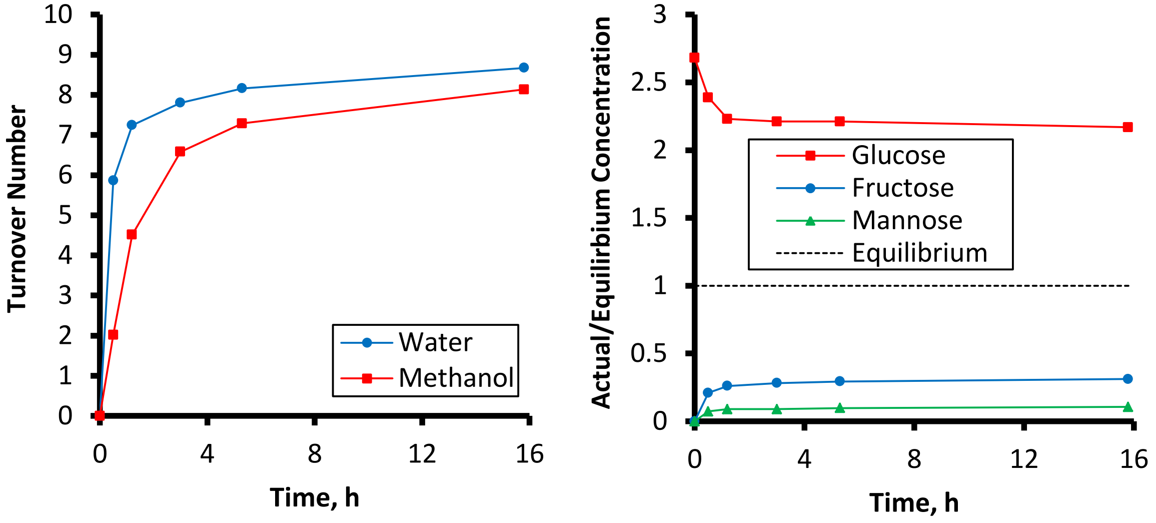

Fig. S6: Left: Stagnation of TON for glucose reactions catalyzed by CIT-6 in water and methanol solvents, based on total Zn content. Right: Approach to equilibrium distribution of sugars in water. Reaction conditions: 100 °C, 1% (wt/wt) glucose, 1:50 Zn:glucose initial molar ratio.

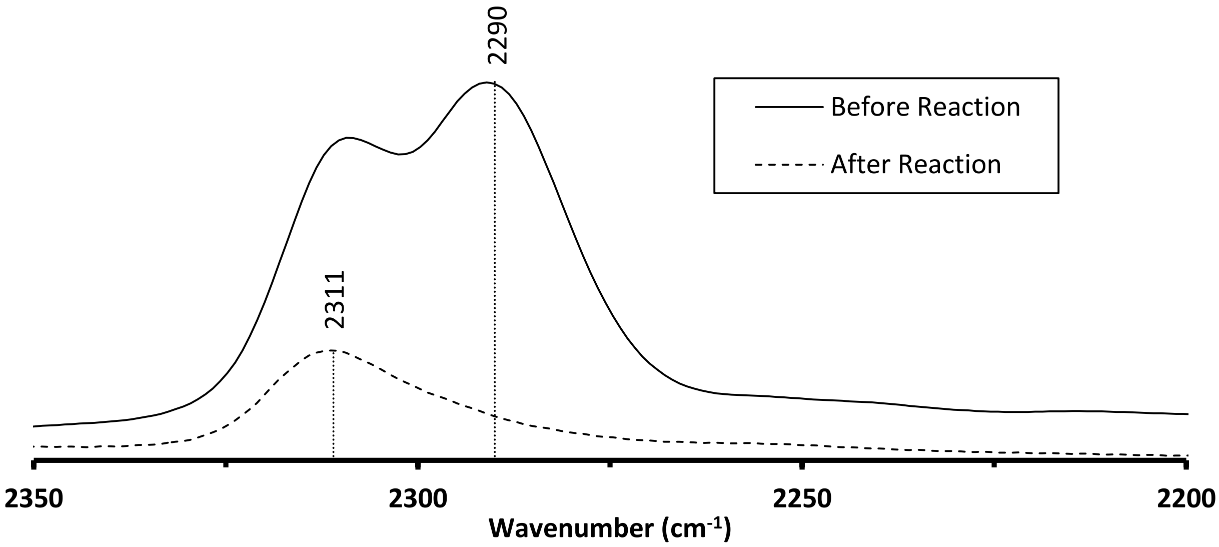

Fig. S7: Baseline-corrected IR spectra of CD_3CN adsorbed at 35 °C on CIT-6 before and after glucose reaction in water. Spectra were collected after desorption at 100 °C for 1h. Spectra are not quantitative and total Zn content (based on EDS measurements) falls by 35% after reaction.

Reaction	Catalyst	Si/M ratio	T (°C)	Time (h)	X_{MMFC} (%)	Y_{MMBC} (%)	S_{MMBC} (%)
1	CIT-6	12.2 ± 0.9	150	12	18.1	10.1	55.7
2	CIT-6	12.2 ± 0.9	170	12	34.5	21.5	62.3
3	CIT-6	12.2 ± 0.9	170	18	45.8	28.4	62.0
4	CIT-6	12.2 ± 0.9	190	6	30.5	15.6	51.0
5	catalyst recovered after **reaction 4** [1]	n.d.	190	6	18.8	10.2	54.4
6	CIT-6	12.2 ± 0.9	190	12	43.6	25.7	58.8
7	CIT-6	12.2 ± 0.9	230	6	41.6	9.9	23.8
8	CIT-6 [2]	12.2 ± 0.9	190	6	*	*	*
9	Bulk ZnO	-	190	6	*	*	*
10	CIT-6-LiEx	10.9 ± 2.5	190	6	4.7	1.6	34.8
11	CIT-6-Z0	21.3 ± 4.2	190	6	51.0	21.9	42.9
12	Zn MCM-41	21.6 ± 8.0	190	6	4.3	*	*
13	Zn MFI	38.8 ± 3.2	190	12	6.8	*	*
14	VPI-8	18.3 ± 1.8	190	6	5.1	*	*
15	SSZ-33-deB-re-Zn-pH=6.9	12.6 ± 0.9	190	12	10.6	0.4	4.1
16	Zn-Na-Al-Beta	**31.0 ± 0.7 Si/Zn**<br>**12.6 ± 0.1 Si/Al**	190	6	89	4.7	5.2
17	CIT-6-de-Zn	188 ± 17	190	6	26.6	3.9	14.7
18	CIT-6-re-Zn-pH=5.1	180 ± 57	190	6	53.1	11.9	22.5
19	CIT-6-re-Zn-pH=6.0	21.8 ± 1.6	190	6	52.5	22.2	42.4
20	CIT-6-re-Zn-pH=6.9	8.5 ± 0.3	190	6	50.7	24.5	48.2
21	CIT-6-re-Zn-pH=7.4	5.9 ± 0.2	190	6	47.0	21.2	44.9
22	Zr-Beta	79 ± 9	190	6	44.7	18.9	42.3

Table S1: Summary of results for DA cycloaddition-dehydration reaction of MMFC and ethylene. Reaction conditions: catalyst, temperature, and catalyst as indicated; 0.1 M MMFC in 10 mL heptane; 100 mg of catalyst; and 35 bar C_2H_2 at 25 °C.

[1] Catalyst washed with heptane, and reused without recalcination

[2] Dioxane solvent (0.1 M MMFC in 10 mL)

* Below detection limit for conversion or yield (0.4%)

n.d. not determined

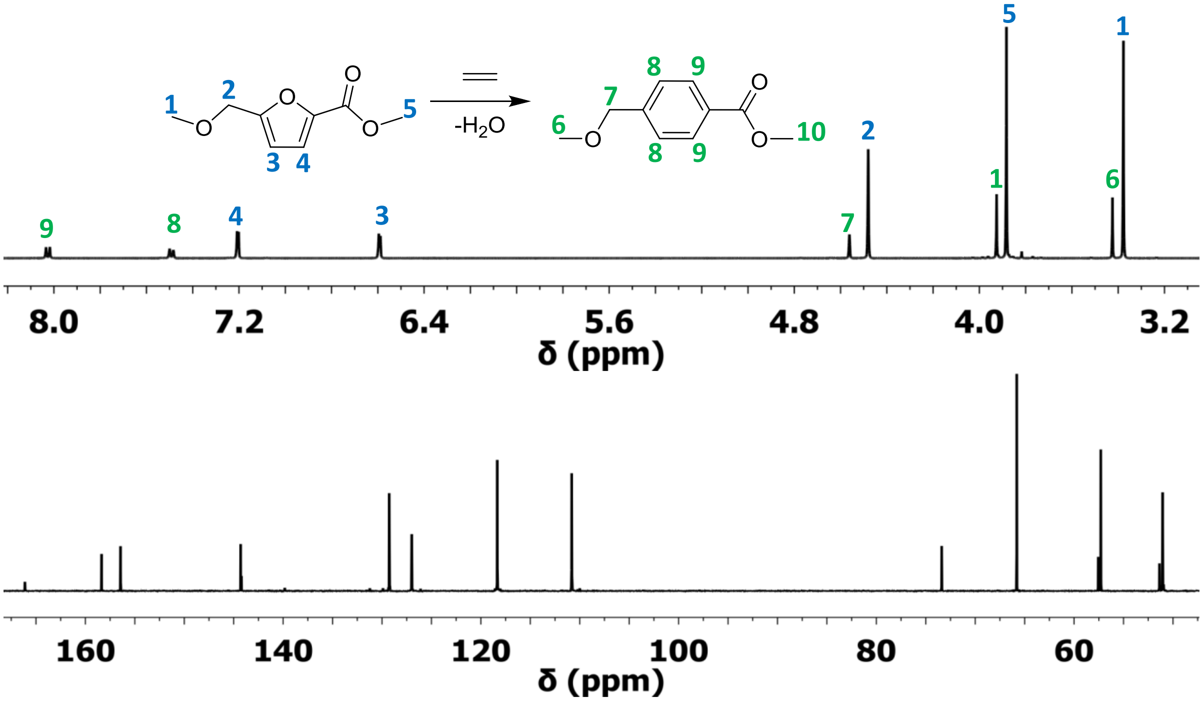

Fig. S8: ^{1}H (top) and ^{13}C (bottom) NMR spectra of unseparated reactant (MMFC) and the products generated by CIT-6 at 190 °C after a 6h DA cycloaddition-dehydration reaction in heptane.

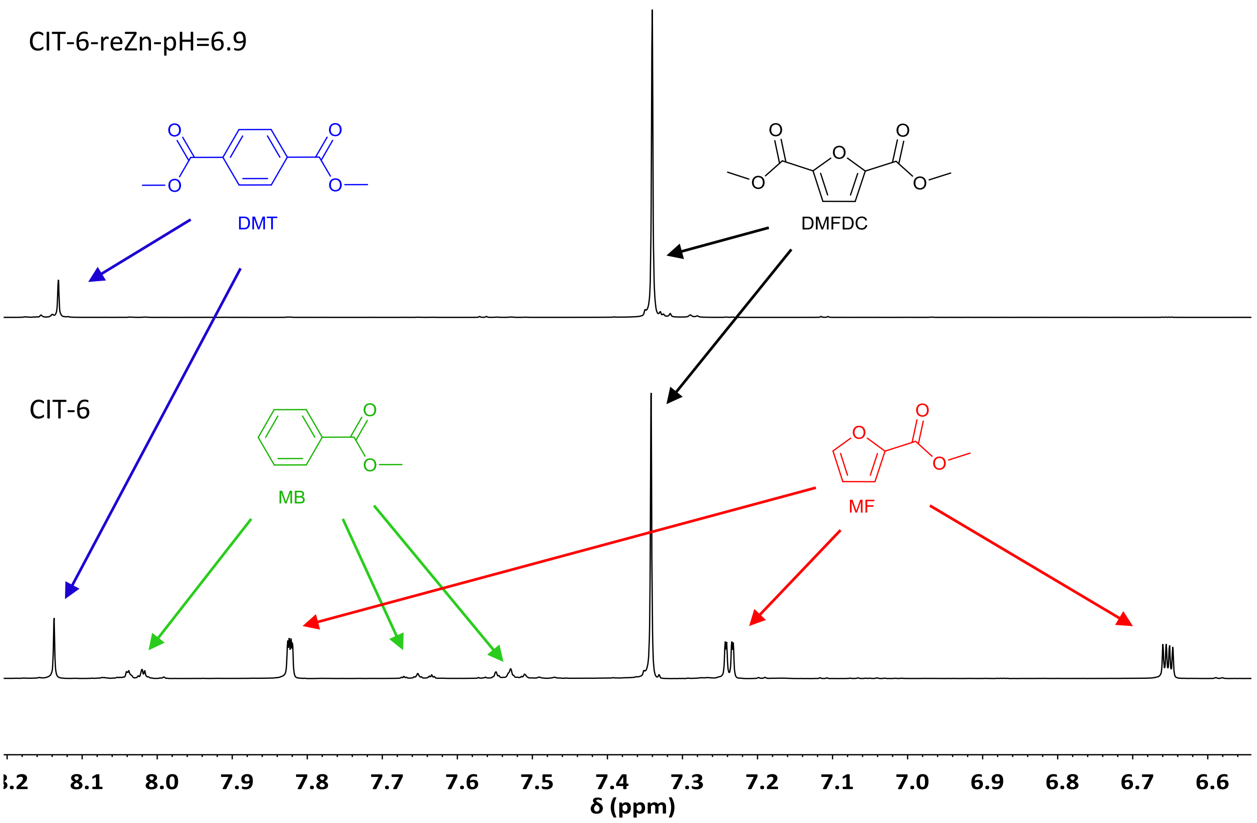

Fig. S9: ^{1}H NMR spectrum of unseparated reactant (DMFDC) and the products (DMT, MB, and MF) generated by CIT-6 (bottom) and CIT-6-reZn-pH=6.9 (top) at 190 °C after a 12h DA cycloaddition-dehydration reaction in heptane.

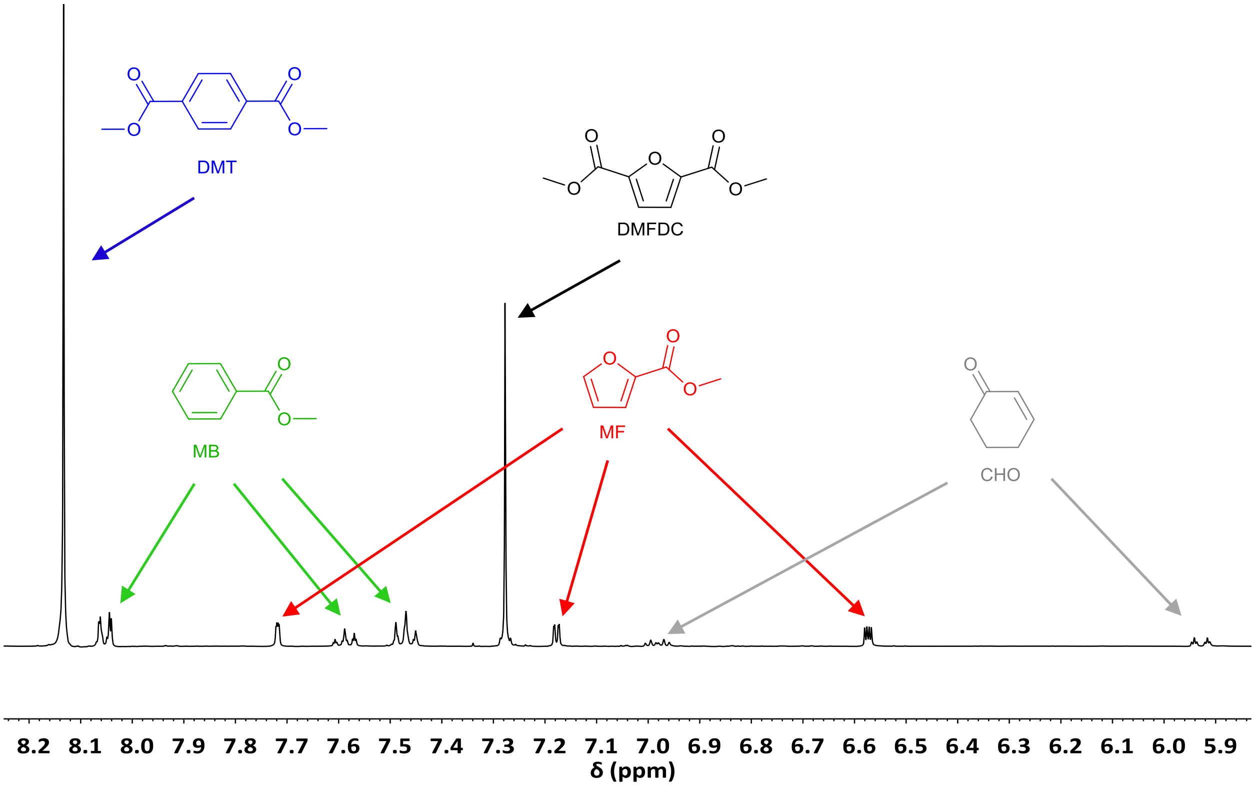

Fig. S10: ^{1}H NMR spectrum of unseparated reactant (DMFDC) and the products (DMT, MB, MF, and CHO) generated by CIT-6-reZn-pH=6.9 at 230 °C after a 12h DA cycloaddition-dehydration reaction in heptane.

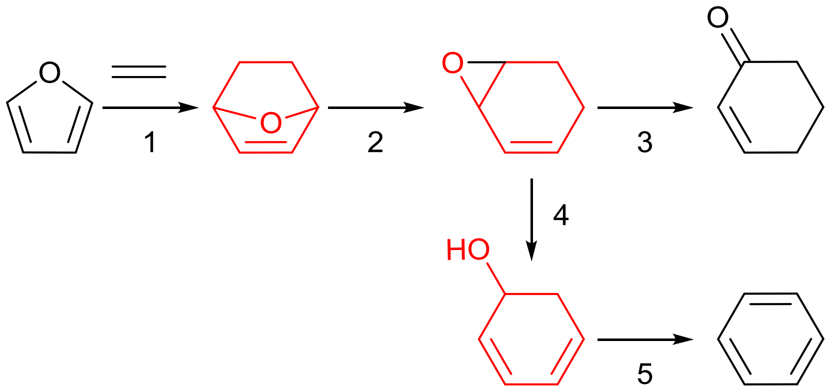

Scheme S2: Proposed mechanism of formation of 2-cyclohexenone and benzene from furan. Step 1 is a Diels-Alder cycloaddition step. Step 2 is a Lewis acid promoted rearrangement to an epoxide proposed for the dimethyl furan analog of the oxa-norbornene cycloadduct. Step 3 is a Lewis acid promoted hydride shift that isomerizes the epoxide to the enone. Benzene is hypothesized to form through steps 4 and 5 that are also proposed for the analogous dehydrative-aromatization of dimethyl furan to p-xylene. Intermediates highlighted in red were not detected in this study.

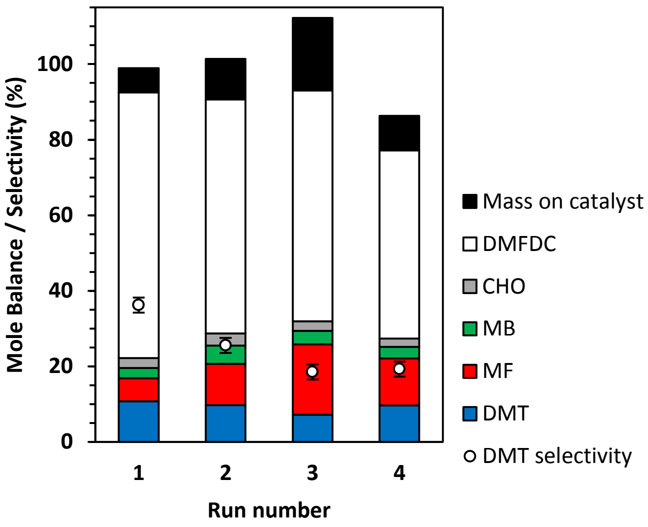

Fig. S11: Catalyst recycle experiments for Diels-Alder cycloaddition-dehydration reactions of DMFDC catalyzed by CIT-6-re-Zn-pH=6.9 at 210 °C. Run 1 uses as-made CIT-6-re-Zn-pH=6.9; Run 2 uses catalyst recovered after Run 1, triply washed by acetone, and dried; Run 3 uses catalyst recovered after Run 2, triply washed by acetone, and dried; Run 4 uses catalyst recovered after Run 3, triply washed by acetone, dried, and calcined. Resulting yields (%) of DMT, MF, MB, CHO, and DMFDC are calculated as ratio of moles formed to initial moles of DMFDC. Mass on catalyst (%) is expressed as ratio of combustible mass on catalyst (measured by TGA) to initial mass of DMFDC. Reaction conditions: reagent and solvent ratios were adjusted to keep constant ratio to inorganic catalyst content between runs; 35 bar C_2H_2 at 25 °C, 12h reaction time.

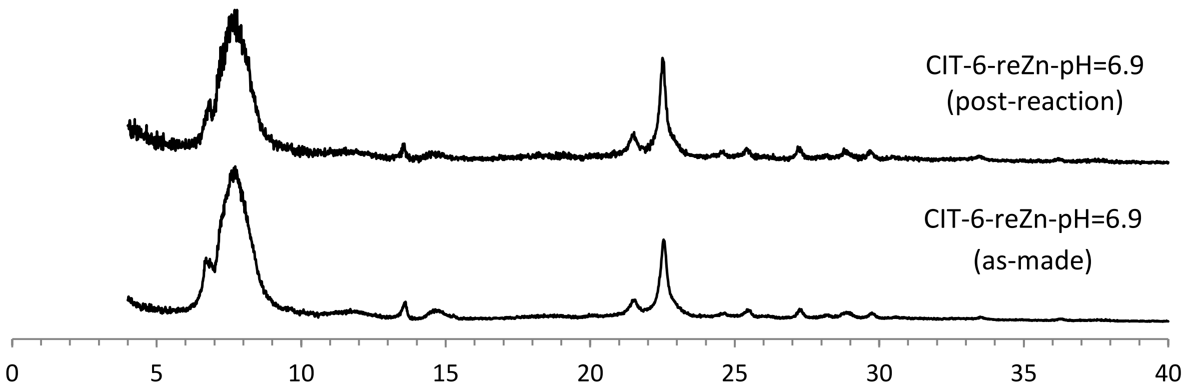

Fig. S12: Normalized powder XRD data for as-made CIT-6-reZn-pH=6.9 (bottom), and CIT-6-reZn-pH=6.9 recovered after Run 3 in Fig. S11 (top).

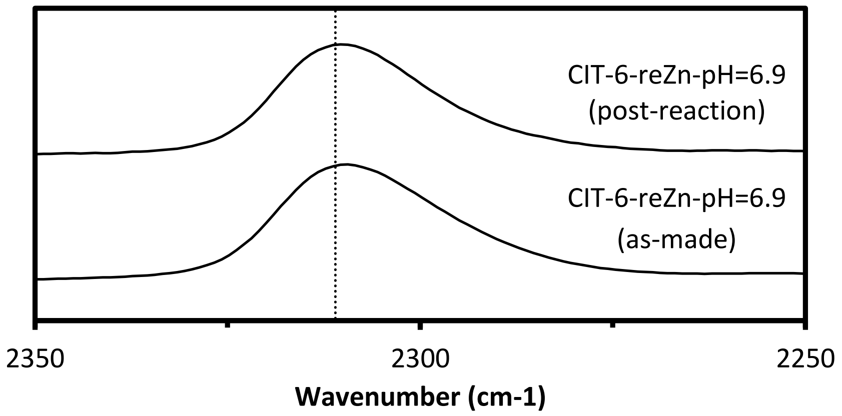

Fig. S13: Baseline-corrected, normalized IR spectra of CD_3CN adsorbed at 35 °C on as-made CIT-6-reZn-pH=6.9 (bottom), and CIT-6-reZn-pH=6.9 recovered after Run 3 in Fig. S11 (top). Spectra were collected after desorption at 100 °C for 1h.

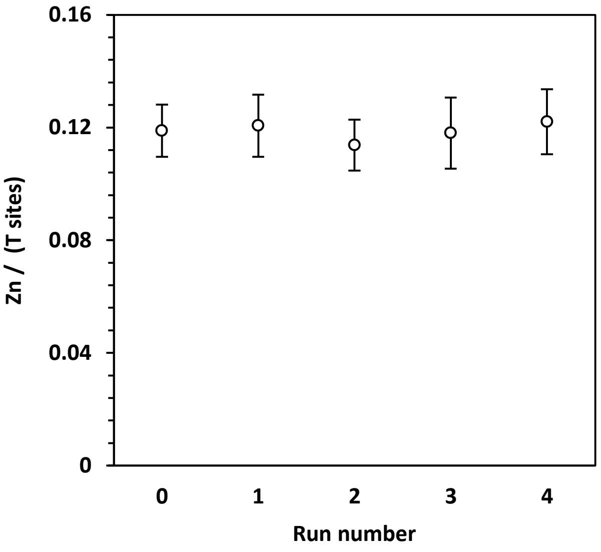

Fig. S14: Zn content (as measured by EDS) of catalysts recovered after each run shown in Fig. S11. Run 0 corresponds to as-made CIT-6-re-Zn-pH=6.9 before Run 1; Run 1 uses as-made CIT-6-re-Zn-pH=6.9; Run 2 uses catalyst recovered after Run 1, triply washed by acetone, and dried; Run 3 uses catalyst recovered after Run 2, triply washed by acetone, and dried; Run 4 uses catalyst recovered after Run 3, triply washed by acetone, dried, and calcined.
